# LncSubpathway: a novel approach for identifying dysfunctional subpathways associated with risk lncRNAs by integrating lncRNA and mRNA expression profiles and pathway topologies

**DOI:** 10.18632/oncotarget.14973

**Published:** 2017-02-01

**Authors:** Yanjun Xu, Feng Li, Tan Wu, Yingqi Xu, Haixiu Yang, Qun Dong, Meiyu Zheng, Desi Shang, Chunlong Zhang, Yunpeng Zhang, Xia Li

**Affiliations:** ^1^ College of Bioinformatics Science and Technology, Harbin Medical University, Harbin, China

**Keywords:** lncRNA, subpathway, network, pathway topologies

## Abstract

Long non-coding RNAs (lncRNAs) play important roles in various biological processes, including the development of many diseases. Pathway analysis is a valuable aid for understanding the cellular functions of these transcripts. We have developed and characterized LncSubpathway, a novel method that integrates lncRNA and protein coding gene (PCG) expression with interactome data to identify disease risk subpathways that functionally associated with risk lncRNAs. LncSubpathway identifies the most relevance regions which are related with risk lncRNA set and implicated with study conditions through simultaneously considering the dysregulation extent of lncRNAs, PCGs and their correlations. Simulation studies demonstrated that the sensitivity and false positive rates of LncSubpathway were within acceptable ranges, and that LncSubpathway could accurately identify dysregulated regions that related with disease risk lncRNAs within pathways. When LncSubpathway was applied to colorectal carcinoma and breast cancer subtype datasets, it identified cancer type- and breast cancer subtype-related meaningful subpathways. Further, analysis of its robustness and reproducibility indicated that LncSubpathway was a reliable means of identifying subpathways that functionally associated with lncRNAs. LncSubpathway is freely available at http://www.bio-bigdata.com/lncSubpathway/.

## INTRODUCTION

LncRNAs are a heterogeneous class of ncRNAs that play key roles in disease development and progression [1] by mediating a variety of biological functions, such as cell differentiation [2], immune responses [3], genomic imprinting [4], and chromatin modification [5]. For example, lncRNAs regulate core elements in the transforming growth factor-β signaling pathway and thus promote tumorigenesis, invasion, and metastasis [6]. Zhang *et al*. demonstrated that the lncRNA CASC11 interacts with hnRNP-K and activates the WNT/β-catenin pathway to promote growth and metastasis in colorectal cancer [7]. However, the mechanisms by which lncRNAs affect disease-associated aberrant pathway activation are not completely understood. Pathway identification may help improve our understanding of the large-scale expression measurements and underlying conditions in these studies.

Many recent studies have investigated the functions of lncRNAs. Several “co-expression-based” methods have been proposed based on the observation that genes with similar expression patterns across multiple experimental conditions may share similar functions or participate in related biological pathways [8, 9]. For example, Guttman *et al*. assigned putative functions to ~1600 lincRNAs identified using chromatin-state maps. Liao *et al*. constructed an lncRNA-protein coding gene co-expression network and used it to predict the functions of the lncRNAs involved [10]. Guo *et al*. provided a global strategy for inferring lncRNA functions in a comprehensive co-expression network [11]. Jiang *et al*. developed the lncRNA2function tool to investigate the function of human lncRNAs based on correlations between their expression and the expression of protein-coding genes across 19 human normal tissues [12]. In addition, Liu *et al*. predicted disease-related lncRNAs based on lncRNA-mRNA co-expression [13]. Instead of using similarities in the expression patterns of lncRNA and protein coding genes, Linc2GO [14] predicted lincRNA functions based on the ceRNA hypothesis, which posits that lncRNAs interact with microRNAs (miRNAs) by acting as sponges. LncRNAs thus indirectly regulate their targets and represent a novel layer of gene regulation that might play critical roles in both physiological conditions and diseases. Sequence-based strategies for examining the relationships between lncRNAs and mRNAs would complement these co-expression-based methods.

Although these methods have been crucial for investigating lncRNA functions and regulation, they were not designed to investigate the functional roles of lncRNAs that contribute to disease states. In addition, most of these methods can predict functions only for individual lncRNAs; however, since single factors alone rarely determine the onset or progression of disease, evaluating sets of risk lncRNAs might be more informative. Multiple risk-associated lncRNAs may collectively impact different, but related, pathways in different conditions [15, 16]. Thus, novel computational methods are needed for functional analysis of lncRNAs. To do this, some important biological aspects should be considered. First, many studies have suggested that abnormalities in “subpathway regions” (i.e. sub-regions within the entire pathway) play important roles in disease etiology [17, 18]. It is therefore possible that lncRNA dysregulation may impact subpathway regions to contribute to disease development. Locating subpathway regions that are associated with dysregulated lncRNAs might help reveal mechanisms by which lncRNAs contribute to disease states. Second, perturbations of signaling pathways that contribute to human diseases can result not only from dysfunctional nodes (e.g. genes or proteins), but also from dysfunctional molecular interactions outside of those nodes [19, 20].

In this study, we propose a novel computational method that integrates transcriptional expression, pathway topologies, and lncRNA-mRNA association network to detect transcriptional subpathway dysregulation that related with dysregulated lncRNAs. We used two distinct but complementary sources of biological data to construct this network: (i) an lncRNA-mRNA co-expression network, which was constructed based on correlations between the expression of lncRNAs and mRNAs from 28 RNA-seq datasets reflecting multiple experimental conditions; (ii) an lncRNA-mRNA association network constructed based on ceRNA theory. We then used the PCST algorithm, which has been used to identify functional modules in protein–protein interaction networks [21–23], to locate dysfunctional pathway regions that were associated with risk lncRNAs; alterations of both PCGs and lncRNAs and the degree of changes in the associations among them were considered simultaneously. Finally, we used random permutation to evaluate each identified subpathway region. We then analyzed data from stimulation, colorectal cancer, and breast cancer studies to demonstrate the effectiveness of our method. We found that LncSubpathway successfully and reliably identified meaningful subpathways related to dysregulated, disease-associated lncRNAs. LncSubpathway is freely available athttp://www.bio-bigdata.com/lncSubpathway/.

## RESULTS

### Simulation I: characteristics of LncSubpathway

We characterized LncSubpathway with respect to changes in the degree to which lncRNAs and PCGs were differentially expressed and interacted in this simulated study. Two simulated pathways (Linear and ERBB) with different patterns of connections between pathway PCGs were examined. To test the effects of increasing the magnitude of changes in nodes (lncRNAs/PCGs) or interactions, simulation datasets were created by varying corresponding parameters.

**Figure 1:**
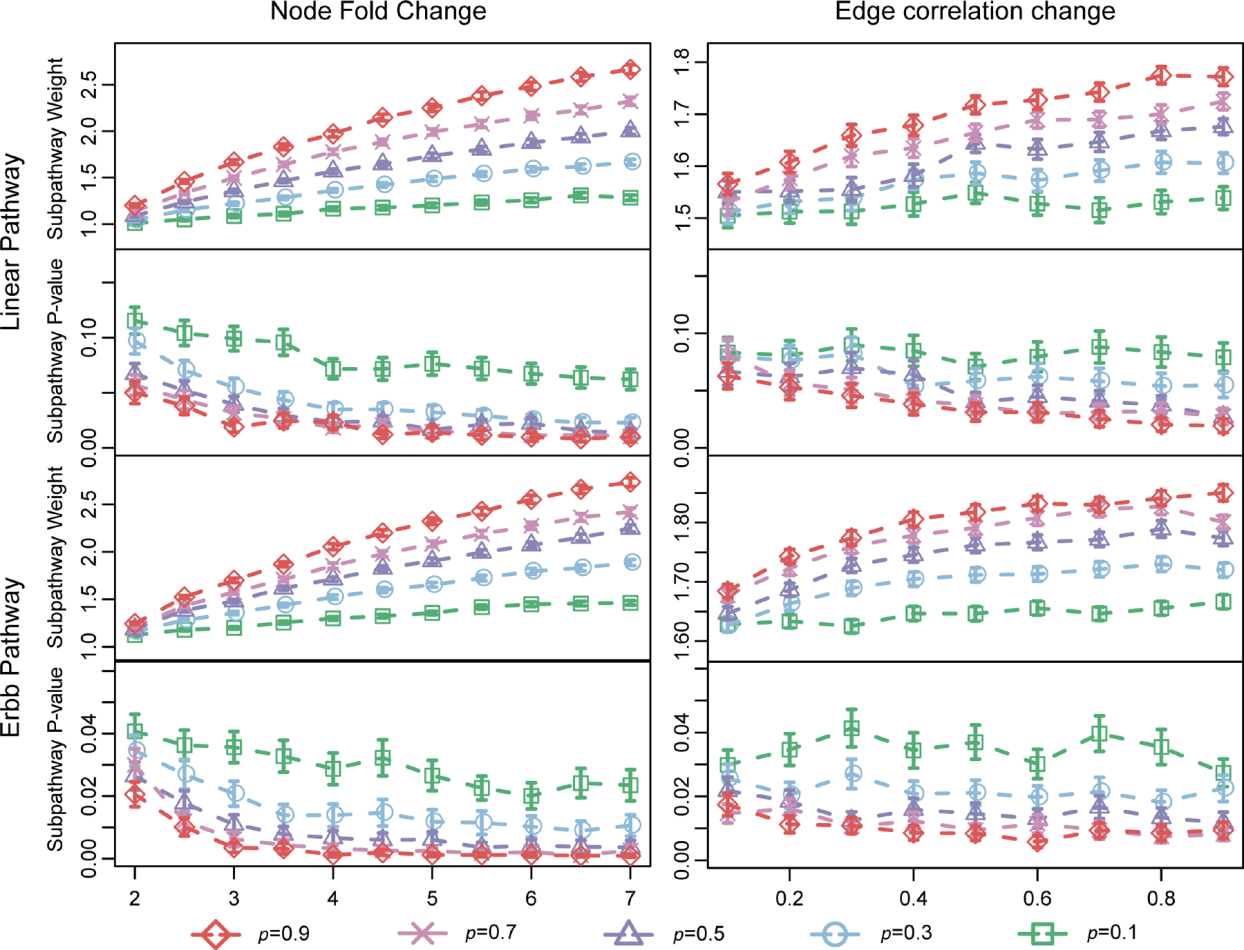
Characteristics of LncSubpathway Subpathway weights and *P*-values obtained when LncSubpathway was used to analyze simulation datasets with different degrees of change for nodes and edges in the linear and ERBB pathway structure models. Node fold-change was varied from 2.0 to 7.0 in increments of 0.5; edge correlation change was varied from 0.1 to 0.9 in increments of 0.1. p, the proportion of pathway-associated nodes (lncRNAs/PCGs) or edges that were changed, varied from 0.1 to 0.9 in increments of 0.2.

Figure 1 shows weights and *P*-values obtained when LncSubpathway was used to analyze the simulated datasets. In general, subpathway weights increased and *P*-values decreased as the extent of the changes in the lncRNAs/PCGs nodes and correlations between them (*n*, *e*, and *p*) increased. In addition, weight values changed similarly when the extent of changes in nodes (lncRNAs/PCGs) or edges was varied (Figure 1), indicating that changes in nodes and interactions contributed equally to weights and *P*-values in the identified subpathways. Furthermore, the *P*-values of subpathways identified in the ERBB pathway were lower than those of the subpathways identified in the linear pathway (Figure 1). This because the ERBB pathway has a more closely-connected structure than the linear pathway, making the formation of a connected subnetwork easier in the ERBB than in the linear pathway when the extent of dysregulation (*n*, *e*, and *p*) is the same.

**Figure 2:**
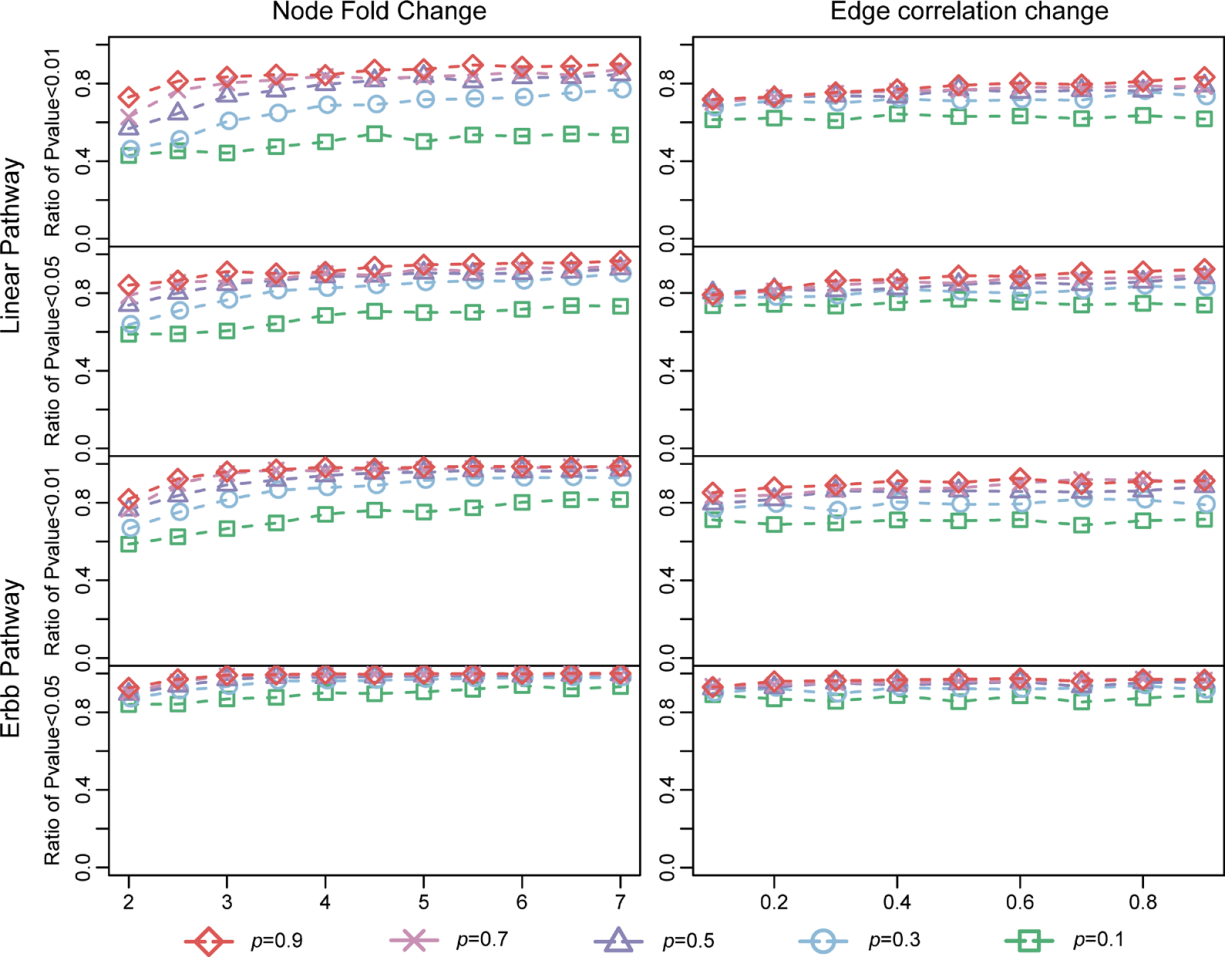
Sensitivity of LncSubpathway The y-axis of each subplot represents the ratio of subpathways that LncSubpathway identified as differential with *P* < 0.01 (*P* < 0.05) after 100 repetitions of the linear and ERBB pathway models. Node (edge) change and the variable *p* were similar to Figure 2.

We then further characterized the sensitivity of LncSubpathway. Ratios of the 100 replicates in which *P* < 0.01 or 0.05 were obtained when LncSubpathway was applied to identify lncRNA-related subpathways for each simulation condition were determined (Figure 2); this ratio was used to measure the sensitivity of LncSubpathway. As shown in Figure 2, in general, the ratio of statistically significant cases increased as the extent of changes increased at both the node (PCG/lncRNA) and edge levels. The sensitivity of LncSubpathway is therefore relatively high under various conditions for these two distinct pathway structure models.

### Simulation II: false positive rates for the LncSubpathway

Due to the high sensitivity of LncSubpathway, it is possible that this method also has a high false positive rate. We therefore used two simulation strategies to analyze the false positive rate of LncSubpathway.

**Figure 3:**
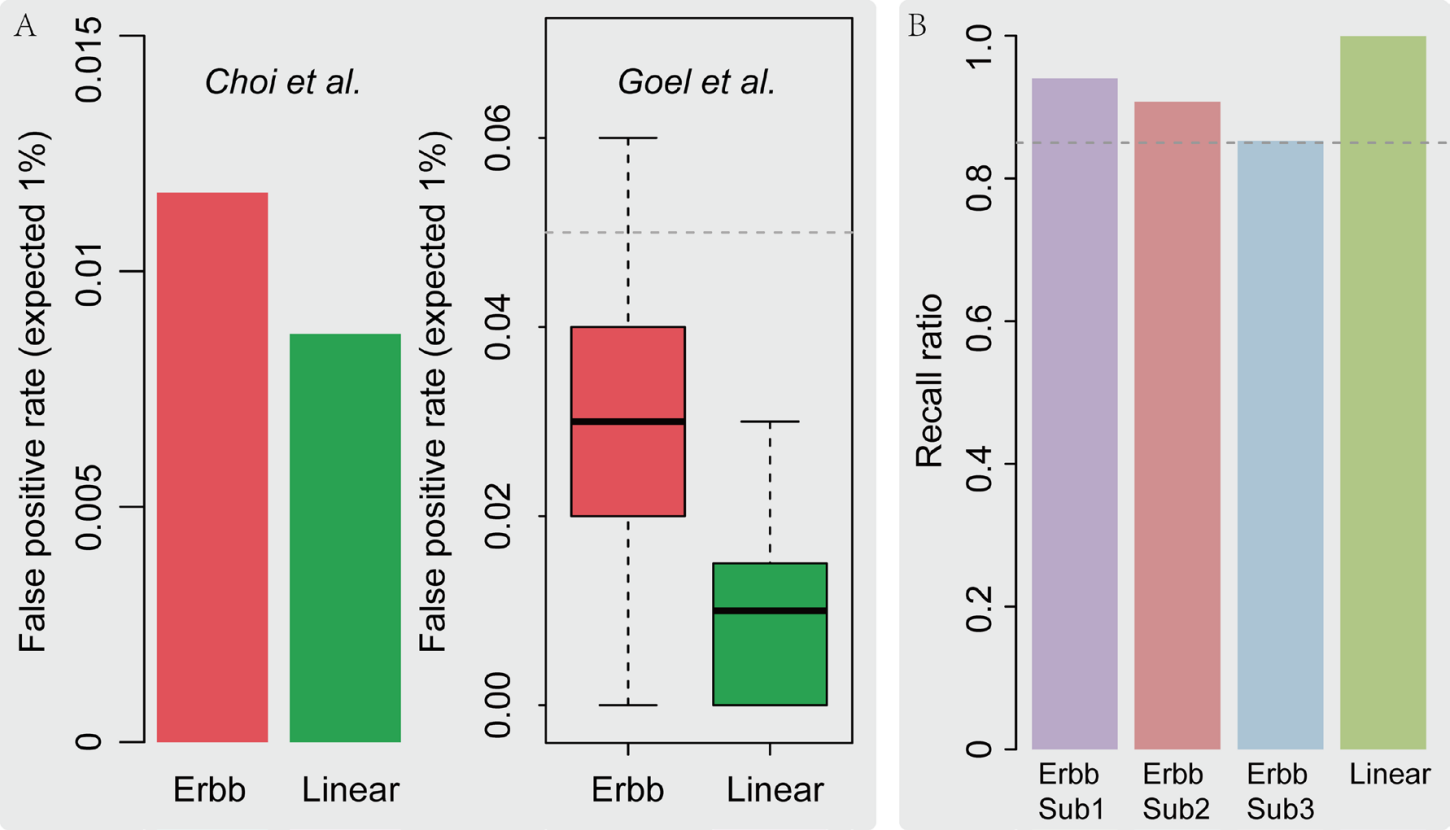
(**A**) False positive rate analysis using simulation datasets. The false positive rate of LncSubpathway evaluated using methods described in Choi et al. (left) and Goel et al. (right) for the Linear and ERBB pathway structure models, respectively. (**B**) The ratio of elements in predefined subpathway regions that were recalled in subpathways identified by LncSubpathway.

Figure 3A shows the evaluation of false positive rates of LncSubpathway, at an excepted rate of 1%, for applying method to simulation datasets that generated according to *Choi et al.’s* method and *Goel et al.’s* method for Linear and ERBB pathway models and sample size 250,300 and 500. The false positive rate of LncSubpathway for these simulated cases was not exceeded 5% (Figure 3A) for both the Linear and ERBB pathway models. This indicates that the false positive rates of LncSubpathway are within an acceptable range.

### Simulation III: the effectiveness of LncSubpathway

To assess the effectiveness of our method, we next examined whether LncSubpathway accurately located dysregulated subpathway regions that were associated with lncRNAs of interest. We assumed that one subpathway region in the linear pathway and three subpathway regions in the ERBB pathway were dysregulated. Simulated datasets were then generated according to the dysregulation patterns of the subpathway regions in Supplementary Figure 1. As shown in Figure 3B, LncSubpathway was highly accurate in identifying all four dysregulated subpathway regions; even the lowest recall ratio value, which was for ERBB subpathway region 3, was still 0.85. This indicates that LncSubpathway is capable of accurately locating dysregulated subpathway regions that are related to lncRNAs of interest.

### Risk lncRNAs related dysregulation subpathways in colorectal cancer

We then used LncSubpathway to identify dysregulated subpathways that were associated with risk lncRNAs in colorectal cancer. Colorectal cancer is well-studied, and many pathways have been reported to be relevance with its development or progression. LncSubpathway identified 27 subpathways (corrected *P* < 0.05) which have at least one lncRNA associate with PCGs within the subpathway. These 27 subpathways correspond to 23 entire pathways. On average, 12.8 lncRNAs and 7.5 key lncRNAs were functionally associated with each subpathway. Among the 27 subpathways identified, up to 21 (78%) have been implicated in the initiation and/or progression of colorectal or other cancers (Suppelmentary Table 1). To examine how these dysregulated subpathways and the related lncRNAs identified by LncSubpathway can provide insight into disease etiology, we examined three representative subpathways, including the p53 signaling pathway (path: 04115_1), the FOXO signaling pathway (path: 04068_1), and purine metabolism (path: 00230_1).

**Figure 4:**
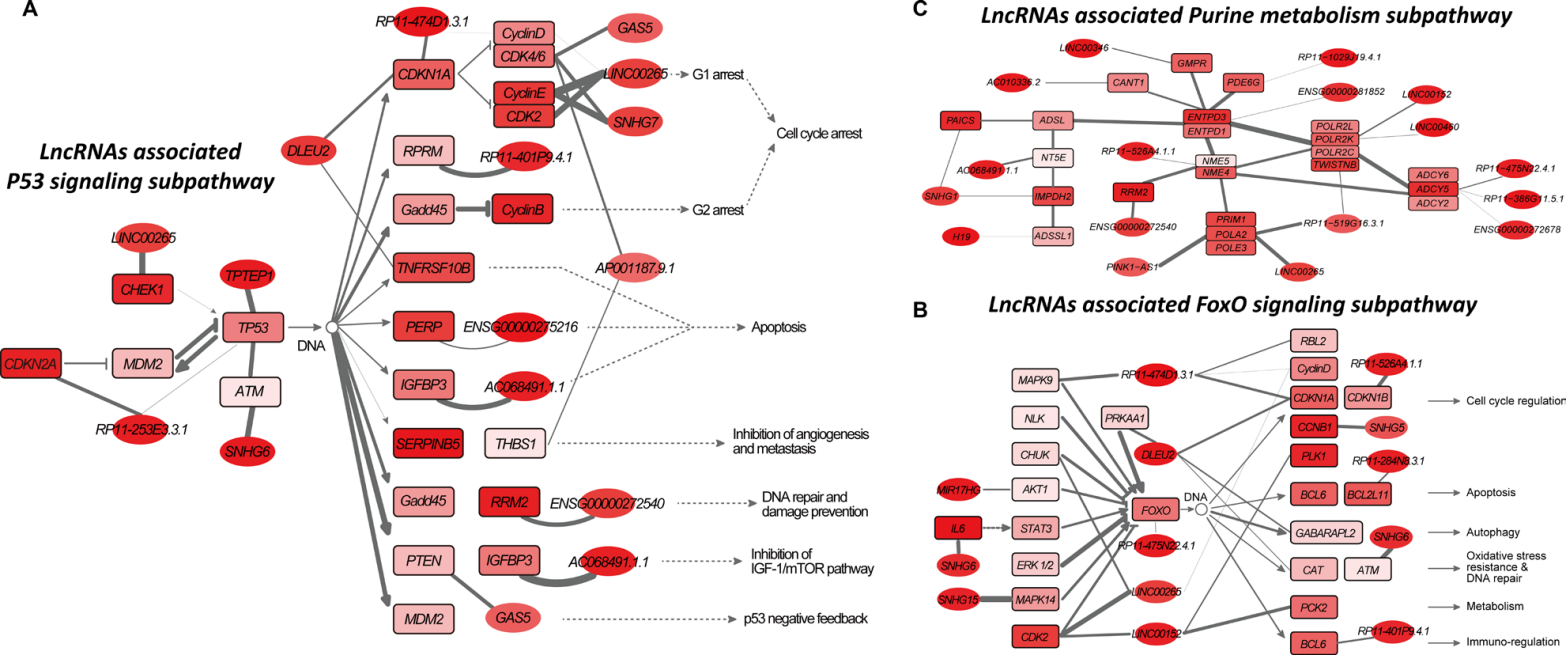
Risk lncRNA-associated subpathways in colorectal cancer Node color is proportional to the differential degree (fold-change value) of lncRNAs/PCGs; edge width corresponds to the degree of change in the correlation. (**A**) Path:04115_1: risk lncRNA-associated subpathway region belonging to the p53 signaling pathway. (**B**) Path:04068_1: risk lncRNA-associated subpathway region belonging to the FoxO signaling pathway. (**C**) Path:00230_1: risk lncRNA-associated subpathway region belonging to purine metabolism.

The first subpathway examined is a TP53-centered subpathway region within the p53 signaling pathway (path: 04115_1) (Figure 4A), which plays a role in the initiation and progression of colorectal cancer. TP53, a well-known tumor suppressor gene that encodes p53 protein, is frequently inactivated by mutations or deletions in most human cancers, including colorectal cancer [24]. For example, p53 is expressed in primary tumors and lymph node metastases in colorectal cancer patients [25]. Furthermore, p53 controls colorectal cancer cell invasion by inhibiting the NF-κB-mediated activation of Fascin [26]. In addition, leukemia inhibitory factor (LIF) inhibits tumor-suppressor p53 via Stat3/ID1/MDM2 in human colorectal cancer [27]. It is worth noting that the interaction between MDM2 and TP53 was involved in the subpathway region identified by LncSubpathway (Figure 4A). We then focused on investigating the relationship between lncRNAs associated with p53 subpathway and colorectal cancer. Growth arrest specific 5 (GAS5), which has been identified as a potential tumor suppressor, is associated with cellular growth arrest and apoptosis processes (https://www.ncbi.nlm.nih.gov). Interestingly, GAS5 was associated with the positive cell cycle regulator CDK6 and thus influenced downstream cell cycle arrest processes in this subpathway region (Figure 4A). Further examination revealed that GAS5 lncRNA may competitively regulate CDK6 via interactions with common miRNAs. In the cell cycle arrest region, GAS5 cooperated with SNHG7, RP11-474D1.3.1, and LINC00265, and its activity was coordinated with cell cycle regulators such as CDK2, CDK4, CDK6, CCND1, CCND2, CCND3, CCNE1, CCNE2, and CDKN1A (p21) (Figure 4A). Together, these results demonstrate that the activity of these lncRNAs and cell cycle regulators is coordinated during colorectal cancer pathogenesis. PTEN, a well-known tumor suppressor that is competitively regulated by GAS5 in the subpathway region, inhibits the cancer-related IGF-1/mTOR pathway. Interestingly, AC068491.1.1, a lncRNA that was upregulated 3.0-fold and with FDR < 0.001, was functionally coordinated with IGFBP3, which is known to be involved in colorectal cancer and liver metastasis [28, 29]. Co-expression correlations based on 28 RNA-Seq datasets confirmed the association between AC068491.1.1 and IGFBP3. The correlation between AC068491.1.1 and IGFBP3 differed between normal and colorectal cancer tumor samples; in normal samples, AC068491.1.1 and IGFBP3 were negatively correlated (r = –0.49, *P* = 0.038), while in colorectal cancer samples they were positively correlated (r = 0.69, *P* = 0.001). This change may be related to the dysregulation of downstream cell growth and apoptosis processes. Together, the above findings suggest that lncRNA AC068491.1.1 is functionally associated with the P53 signaling pathway and may thus play a critical role in colorectal cancer.

**Figure 5:**
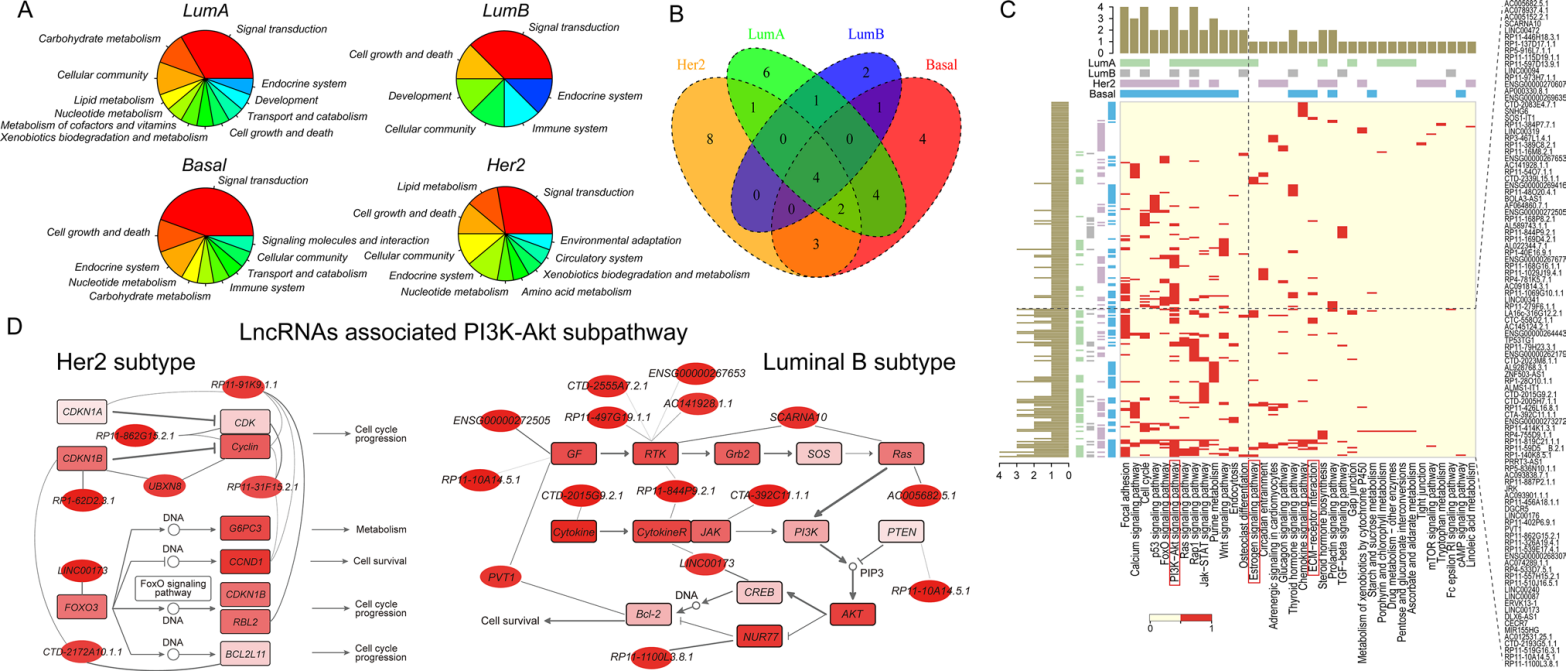
Identification of subpathways associated with risk lncRNAs in breast cancer subtypes (**A**) The distribution of significant subpathways identified by LncSubpathway for each subtype based on relevant functional groups. (**B**) Venn diagram plot of significant subpathways identified by LncSubpathway for each subtype based on the entire pathway to which they belonged. (**C**) Global view of key lncRNA-associated pathways across four breast cancer subtypes. Red in the heatmap represents lncRNAs functionally associated with the corresponding pathway. Bars represent the number of subtypes with which the corresponding key lncRNA (pathway) was associated. (**D**) Two PI3K-Akt subpathway regions functionally associated with risk lncRNAs in HER2 and luminal B subtypes. Node color is proportional to the fold-change value of lncRNAs/PCGs; edge width corresponds to the degree of change in the correlation. Left: path: 04151_6 for HER2 subtype; right: path: 04151_1 for luminal B subtype.

The second subpathway we explored is the FOXO signaling subpathway (Figure 4B), which was identified as a significant subpathway due to dysregulation at the node level (*P* < 0.001), but not the edge level (*P* = 0.344) (Supplementary Table 1 and Supplementary Figure 2). Notably, the expression of the key lncRNAs associated with this subpathway changed nearly 4-fold on average. The transcription factor FOXO has been considered a tumor suppressor that limits cell proliferation and induces apoptosis [30] and also regulates energy metabolism and development in several tissues [31]. FoxO3A, a member of the FOXO transcription factor family, is modulated by AMPK. The AMPK-FoxO3A axis is activated in colorectal cancer cell and may be a promising therapeutic target [31]. Interestingly, the AMPK (PRKAA1)-FoxO3A axis was centrally located in the subpathway region identified by LncSubpathway (Figure 4B). In determining how dysregulation of the lncRNAs associated with this subpathway is implicated in colorectal cancer pathogenesis, we first noted that the lncRNA DLEU2 (fold-change > 2) competitively regulated AMPK (PRKAA1); the ceRNA dataset supported this association. The dysregulation of DLEU2 may be associated with the AMPK-FoxO3A axis and thus promote uncontrolled cell growth in colorectal cancer. Interestingly, in addition to upstream PRKAA1, DLEU2 was also associated with multiple downstream factors, including CDKN1A, GABARAPL2, and CAT, and might therefore also impact cell cycle, autophagy, oxidative stress, and DNA repair functions (Figure 4B). In addition, we found that RP11-474D1.3.1, lncRNA with the largest expression change in the FOXO signaling pathway, competitively regulated the MAPK9, RBL2, CDKN1A, and CCND2 genes, thus influencing cell cycle regulation (Figure 5B). Next, we identified three miRNAs, hsa-miR-106b, hsa-miR-17, and hsa-miR-20a, that were shared by the regulatory relationships between RP11-474D1.3.1 and MAPK9, RBL2, CDKN1A, and CCND2. The role of these three miRNAs in colorectal cancer is well-documented, indicating that the highly dysregulated RP11-474D1.3.1 lncRNA plays an important role in colorectal cancer.

Finally, we examined a purine metabolism subpathway which was identified as significant mainly due to dysregulation of the edges (Figure 4C). The differences in correlation for the edges in this subpathway region were higher than those for the background (Supplementary Figure 3). Purine metabolism affects tumor progression. For example, purine-metabolizing ectoenzymes mediate the production of IL-8, which plays important roles in both diseases related to chronic inflammation and tumor modulation in human colon HT-29 cells [32]. We found that H19, a cancer lncRNA that is associated with many cancer types, including colorectal cancer [33, 34], was functionally associated with the purine metabolism subpathway. Moreover, some PCGs, such as IMPDH2 [35, 36], RRM2 [37–39], and PAICS [40], involved in this subpathway region are closely associated with colorectal or other types of cancer. The lncRNA SNHG1, which is involved in several cancers, such as hepatocellular carcinoma [41] and non-small cell lung cancer [42], was functionally associated with PAICS and IMPDH2 in this subpathway. Although the role of SNHG1 in colorectal cancer remains largely unknown, our results indicate that it may be important in tumorigenesis and progression. The above results suggest that LncSubpathway can identify risk lncRNAs functionally related subpathway regions that were dysregulated at the edge level.

In summary, the above results demonstrated that LncSubpathway is effective in locating risk lncRNA-associated subpathway regions with dysregulation at the node or/and edge levels. LncSubpathway might therefore help identify the functional roles of lncRNAs and novel lncRNAs underlying diseases.

### Identifying risk lncRNA-associated subpathways provides novel insights into breast cancer subtypes

In this section, we examined the ability of LncSubpathway to provide information regarding differences between disease subtypes. We applied LncSubpathway to the breast cancer subtype dataset and identified risk lncRNA-associated subpathways for each subtype (luminal A, luminal B, HER2, and basal) (Supplementary Tables 2, 3, 4, 5). Supplementary Tables 2, 3, 4, 5 show dysregulated subpathways which have at least one lncRNA associate with PCGs within them for each subtypes. Figure 5A shows the biological functions to which the subpathways identified using LncSubpathway contribute for each subtype (FDR < 0.05). In general, all four breast cancer subtypes were associated with subpathways involved in generic cancer-related biological functions, such as signal transduction, cell growth and death, and cellular community. This indicates that risk lncRNAs associated with different subtypes may participate in similar cancer-related functions. In addition, some functions were identified that were associated with specific subtypes. Subpathways related to signaling molecules and interactions were specifically identified for the basal-like subtype. This is consistent with the clinical characteristics of the basal-like subtype, which has high rates of recurrence and metastasis, with which signaling molecules and interaction pathways are closely associated. In addition, lipid metabolism was associated with the luminal A and HER2 subtypes, but not with the other two subtypes. While elevated levels of STAR-related lipid transfer protein 3 may contribute to progression of HER2-positive breast cancers [43], the contributions of lipid metabolism abnormalities to progression in the luminal A subtype requires further study. The above findings suggest that LncSubpathway can identify unique risk lncRNA-related functional groups that correspond to the clinical and molecular characteristics of different breast cancer subtypes.

We then explored the entire pathways to which these risk lncRNA-related, subtype-associated subpathways belonged. As shown in Figure 5B–5C, some generic cancer pathways, such as cell cycle, focal adhesion, and PI3K-Akt signaling pathways, were associated with all four subtypes, while some pathways were subtype-specific. Two notable examples of subtype-specific pathways are the estrogen signaling pathway for the luminal A subtype and the ECM-receptor interaction pathway for the basal-like subtype (Figure 5C). The estrogen signaling pathway plays an important role in the development and treatment of the luminal A subtype, which is estrogen receptor-positive. Meanwhile, the ECM pathway is closely related to cancer cell invasion; invasive and metastatic cells must cross the basement membrane's extracellular matrix to disseminate to distant sites [44–46], and basal-like breast cancer is characterized by high levels of invasion and metastasis. The above results indicate that the dysregulation of lncRNAs with different functions may contribute to the development of different disease subtypes.

While PI3K-Akt subpathways were identified in both the HER2 and luminal B subtypes, the specific sub-regions within the entire PI3K-Akt pathway that were dysregulated differed between the two subtypes (Figure 5D). Dysregulation of the PI3K-Akt pathway is closely related to the initiation and development of breast cancer [47]. CDK, a well-known cancer driver gene, was involved in the Her2-related subpathway region (Supplementary Figure 4). Goel *et al*. demonstrated that CDK4/6 inhibitors could overcome therapeutic resistance in HER2 breast cancer [48]. In contrast, the Luminal B subtype-related region included the Ras-PI3K pathway (Supplementary Figure 4); Ras, in combination with the oncogenic mutant form of PIK3CA, induces metastasis in luminal B subtypes [49]. LncRNA PVT1 was also functionally associated with the luminal B-related region (Figure 5D). Several studies have demonstrated the important roles of PVT1 in breast cancer [50, 51]. In particular, Zhang *et al*. found that aberrant PVT1 expression is associated with the proliferation of breast cancer cells [52]. Interestingly, PVT1 was functionally associated with VEGFA (GF) and Bcl-2 and may thus impact downstream cell proliferation and apoptosis in the luminal B-related region (Supplementary Figure 4). Furthermore, these lncRNAs that are functionally related to different subpathway regions may play specific roles in the corresponding subtypes. Together, the above findings suggest that LncSubpathway can also precisely identify disease subtype-specific, risk lncRNA-related subpathways.

### Reproducibility and robustness of LncSubpathway

**Figure 6:**
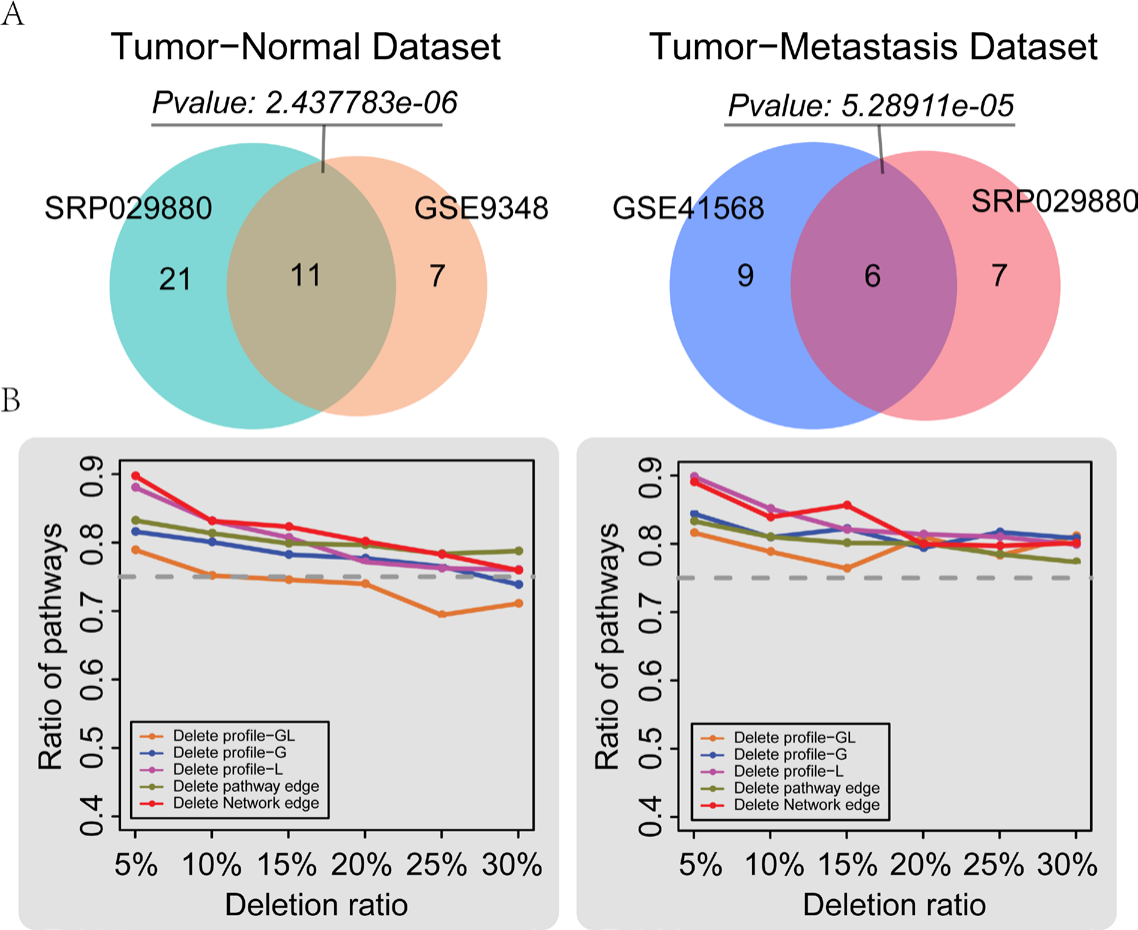
Reproducibility and robustness analyses (**A**) Reproducibility of LncSubpathway. Left: comparison of subpathways identified based on the tumor and normal subsets of the SRP029880 and GSE9348 datasets. Here, 32 entire pathways contain all significant subpathways for SRP029880 and 18 entire pathways contain significant subpathways which have at least one lncRNA associate with PCGs within them for GSE9348 were compared. Right: comparison of subpathways which have at least one lncRNA associate with PCGs within them identified based on the tumor and metastasis subsets of the SRP029880 and GSE41568 datasets. (**B**) Robustness of LncSubpathway. The mean ratio of recalled pathways after *n*% of lncRNAs (PCGs) in the expression profiles, *n*% of edges within pathways, or *n*% edges in the lncRNA-PCG association network were randomly deleted; n varied from 5 to 30 in increments of 5. Left: robustness analysis based on the tumor and normal subset of SRP029880. Right: robustness analysis based on the tumor and metastasis subset of SRP029880. Deletion profile GL: lncRNA and PCG profiles were simultaneously deleted; Deletion profile G: only the PCG profile was deleted; Deletion profile L: only the lncRNA profile was deleted.

To evaluate the reproducibility of LncSubpathway, we used two additional colorectal cancer databases that included primary tumor and normal samples from the GSE9348 dataset and primary tumor and metastasis samples from the GSE41568 dataset. We re-annotated these two expression profiles to obtain sample-matched lncRNA and mRNA profiles. Using LncSubpathway, we then analyzed the two re-annotated datasets and two subsets of the RNA-Seq dataset SRP029880, which was used in the above analysis comparing tumor samples against normal and metastasis samples. LncSubpathway identified 21 significant subpathways (FDR<0.05) which have at least one lncRNA associate with PCGs within them corresponding to 18 entire pathways for the GSE9348 dataset and 39 significant subpathways (FDR<0.05) corresponding to 32 entire pathways for the primary tumor vs. normal sample SRP029880 dataset. Among the 18 pathways identified in the GSE9348 dataset, up to 11 (61.1%) were also identified in these 32 entire pathways of SRP029880. This pathway overlap was statistically significant (*P* = 2.44e–06, hypergeometric test) (Figure 6A). Furthermore, the overlap of entire pathways which contain significant subpathways that have at least one lncRNA associate with PCGs within them for SRP029880 and GSE9348 was also significant. Similarly, the overlap in pathways identified in the two tumor vs. metastasis datasets was also statistically significant (*P* = 5.29e–05, hypergeometric test) (Figure 6A). These results indicate that LncSubpathway generated reproducible results and that it is reliable for the integrative analysis of lncRNA and mRNA expression at the subpathway level.

Biological or measurement-related noise may exist in the expression and biological network data examined here. To evaluate whether LncSubpathway was sensitive to this noise, we performed removal perturbation experiments on the lncRNA and/or mRNA expression profiles, pathway structures, and lncRNA-mRNA association networks for the tumor-normal and tumor-metastasis subsets of the SRP029880 dataset. Specifically, we randomly deleted n% of the lncRNAs or/and mRNAs from the expression profiles, n% of the edges within the pathways, and *n*% of the associations in the lncRNA-protein coding gene network, respectively. For each deletion type, n was set at 5, 10, 15, 20, 25, or 30; the deletion process was repeated 100 times for each scenario. LncSubpathway was used to analyze each dataset generated by this random deletion, and the ratio of identified subpathways that were identified in the original pathway list at an FDR < 0.05 significance level was determined. Overall, the ratio of pathway overlap decreased as the deletion proportion increased for both datasets (Figure 6B). However, the recalled pathway ratio was higher than 75% in most of the deletion cases, except for those in which more than 15 percent of the lncRNA and mRNA profiles were simultaneously deleted from the normal vs. tumor dataset (Figure 6B). We then further explored the overlap pathway ratio from a rank point of view. The top 20 pathways from the original pathway list and from the pathway list generated by random deletion were compared. The results were consistent with the above deletion analysis; specifically, even when the deletion rate was increased to 30%, the pathway overlap ratios remained above 60% in most cases (Supplementary Figure 5). Taken together, the above results suggest that LncSubpathway was robust in resisting disturbances in expression profiles, pathway structures, and lncRNA-mRNA association networks.

## DISCUSSION AND CONCLUSIONS

Thousands of lncRNAs that might regulate a variety of biological processes and play critical roles in disease have already been identified, and the list continues to grow. However, most lncRNAs have not been functionally characterized, and their roles in diseases remain unclear. Identification of functional relationships between lncRNAs and disease-relevant subpathways might help to characterize the effects of lncRNAs in both normal biological phenomena and human diseases. Here, we developed the LncSubpathway method, which identifies lncRNAs associated with transcriptional dysregulation within pathways by integrating lncRNA-mRNA expression and pathway topologies. LncSubpathway simultaneously considers the degree of dysregulation of PCGs and edges within a pathway and changes in lncRNA expression and in correlations between lncRNAs and PCGs. First, we evaluated the characteristics and accuracy of LncSubpathway in three simulation experiments. The first simulation experiment characterized the sensitivity of LncSubpathway with respect to changes in the extent of differential lncRNA/PCG expression and in the extent to which their interactions were differential. The results indicate that LncSubpathway is sensitive to changes in the degree of differences in lncRNA/PCG expression and in their correlation with each other. We also found that the sensitivity of LncSubpathway is relatively high. The second simulation experiment evaluated false positive rates associated with LncSubpathway; the false positive rates were relatively low and within an acceptable range. The third simulation experiment examined whether LncSubpathway accurately and effectively located dysregulated regions that were associated with lncRNAs of interest. The results indicated that LncSubpathway performed well in this regard. Furthermore, when LncSubpathway was used to analyze the colorectal cancer and breast cancer datasets, it successfully identified subpathway regions that were functionally consistent with known risk lncRNAs. For example, LncSubpathway located cell cycle arrest-related subpathway regions that were associated with the lncRNA GAS5, a potential tumor suppressor associated with cellular growth, arrest, and apoptosis processes. LncSubpathway similarly identified subpathways with molecular characteristics that were consistent with specific breast cancer subtypes.

We constructed lncRNA-coding gene associations by integrating complementary co-expression-based and sequence-based association datasets. In order to ensure the reliability of the association network, lncRNA-coding gene pairs in the co-expression network were required to be significantly co-expressed in at least 3 of the 28 RNA-seq datasets. This criterion has been used in previous studies of co-expression among lncRNAs and genes [8, 11]. In addition, lncRNA-coding gene associations inferred from sequence similarity were evaluated using both hypergeometric tests and Jaccard Coefficients. Because the lncRNA-coding gene association network was constructed using computational methods that might introduce false positive associations, we further tested LncSubpathway by randomly removing 5%, 10%, 15%, 20%, 25%, or 30% of the associations from the original network. The results indicated that LncSubpathway was robust; false positive rates remained relatively low even when tested under high disturbance conditions. As lncRNA target gene identification technology continues to improve, the numbers of experimentally identified or computationally predicted lncRNA-mRNA interactions, such as those predicted using the LncTar tool [53], will continue to grow. These associations are also feasible for LncSubpathway. We will also consider the positive or negative regulation of lncRNA on pathway in the future study.

Several methods and tools, such as lncRNA2function [12], Linc2GO [14], lncRNAtor [54] and Co-LncRNA [55], have previously been used to investigate lncRNA functions. Most of these methods can provide functional contexts only for individual lncRNA, while Co-LncRNA can evaluate the combinatorial effects of a maximum of three lncRNAs. However, multiple lncRNAs can cooperate to impact disease development and progression [15, 16]. Thus, the ability to investigate the functional roles of large lncRNA sets could help improve our understanding of the biological phenomena underlying various diseases. Furthermore, previous methods have not considered pathway topological information, which is important for functional analysis. LncSubpathway identifies transcriptionally dysregulated subpathway regions that associated with risk lncRNAs by integrating lncRNA-mRNA expression and pathway topologies. In addition, the degree of dysregulation of the lncRNAs, PCGs, and correlations between them were also considered in our approach. Additionally, LncSubpathway can provide more detailed information regarding lncRNA-related transcriptional dysregulation, such as dysregulation of interactions associated with risk lncRNAs, than the other methods.

Another advanced feature that distinguishes LncSubpathway from previous methods is that it provides relevant functional contexts for risk lncRNAs at the subpathway level. Several studies have demonstrated that abnormalities in subpathway regions may be associated with diseases [17, 18, 56]. LncSubpathway can provide more detailed information about lncRNAs that are associated with transcriptional dysregulation. Interestingly, LncSubpathway identified different dysregulated subpathway regions within the same overall pathway for different breast cancer subtypes (HER2 and luminal B), and the subpathway regions identified corresponded to specific molecular and clinical characteristics of each subtype. This indicates that the high-resolution LncSubpathway method can provide novel insights into the molecular mechanisms underlying specific disease subtypes.

Taken together, our findings demonstrate that LncSubpathway identified biologically meaningful, risk lncRNA-associated subpathway regions for both diseases and disease subtypes. LncSubpathway may therefore improve our understanding of the functional roles of lncRNAs and help to characterize the biological phenomena underlying various diseases.

## MATERIALS AND METHODS

### Data sets

### RNA-seq datasets for constructing the global lncRNA-mRNA co-expression network

We downloaded 28 human RNA-Seq datasets generated under different experimental conditions from the NCBI Sequence Read Archive (SRA) databases [57] (Supplementary Table 6) which were used to construct the lncRNA-mRNA co-expression network. All of these datasets had previously been used by Li *et al*. to predict isoform functions based on an isoform co-expression network [58]. Each data set contains at least six experiments; none of them were population studies. We downloaded lncRNA and protein coding gene annotations from the GENCODE database (http://www.gencodegenes.org/). For each dataset, we aligned the RNA-seq reads of these samples to the human genome (GRCh38) using TopHat (V2.0.13) [59, 60] and then used Cufflinks (V2.2.1) [61] to evaluate the expression of lncRNAs and protein coding genes.

### Colorectal cancer datasets

(1) Colorectal cancer dataset 1: we obtained the RNA-seq dataset for colorectal cancer (SRP029880) from Kim *et al*.'s study, which contains 54 samples (normal colon, primary colorectal cancer, and liver metastases) collected from 18 colorectal cancer patients [62] .The expression of lncRNAs and protein coding genes was quantified using both the TopHat [60] and Cufflinks [61] RNA-seq data processing tools. Matched lncRNA-mRNA expression profiles were filtered to include only profiles with non-zero lncRNA/mRNA expression values in at least 20% of the samples. We used both the DEGSeq [63] and fold-change (FC) methods to identify differentially expressed lncRNAs, which were designated risk lncRNAs. A lncRNA was considered differentially expressed when it was identified as significant using the DEGSeq method (FDR < 0.25) and had an FC value of either > 1.5 or < 2/3. (2) Colorectal cancer dataset 2: the gene expression profile for colorectal cancer from Hong *et al*.'s study, which includes 70 tumor samples and 12 healthy controls, was downloaded from the GEO database (GSE9348). We re-annotated the probes corresponding to protein coding genes and lncRNAs in the microarray using strategy similar to the computational pipeline of Liao *et al*. [10]. Using this re-annotation strategy, we obtained matched sample expression profiles for both lncRNAs and protein coding genes. Differentially expressed lncRNAs were identified using both *t*-tests and the FC method. A lncRNA was considered differentially expressed when it was identified as significant by the *t*-test method (FDR < 0.25) and had an FC value of either > 1.5 or < 2/3. (3) Colorectal cancer dataset 3: the gene expression profile for colorectal cancer including both tumor and metastasis samples was downloaded from the GEO database (GSE41568). Differentially expressed lncRNAs were identified using *t*-tests (FDR < 0.25), and the re-annotation pipeline was used as described for colorectal cancer dataset 2.

### Breast cancer subtype dataset

We downloaded level 3 RNA (Illumina-HiSeqRNASeqV2) expression data for breast cancer from the TCGA database (version: April, 2015) through the Data portal (http://cancergenome.nih.gov/). We then extracted protein coding gene expression data for each sample as described in our previously study [64]. The TCGA breast cancer sample lncRNA expression data were obtained from Li *et al*. [65]. Samples for which both lncRNA and mRNA expression were available were retained in the analysis. Breast cancer samples were assigned to either the Luminal A, Luminal B, Her2-enriched, or Basal-like subtypes according to the guidelines in Ciriello *et al*. [66] using a 50-gene signature (PAM50)-based subtype classification. Ultimately, we obtained matched lncRNA and protein coding gene expression profiles for 232 luminal A, 110 luminal B, 40 HER2-enriched, and 77 basal-like samples. The matched breast cancer lncRNA-mRNA expression profiles were filtered using the same method applied to the colorectal cancer dataset. Differentially expressed lncRNAs were obtained for each subtype by comparing lncRNA expression for samples with that subtype to samples belonging to the other three subtypes using both DEGSeq [63] and FC methods. A lncRNA was considered differentially expressed when it was identified as significant with the DEGSeq method (FDR < 0.25) and had an FC value of either > 1.5 or < 2/3.

### Methods

**Figure 7:**
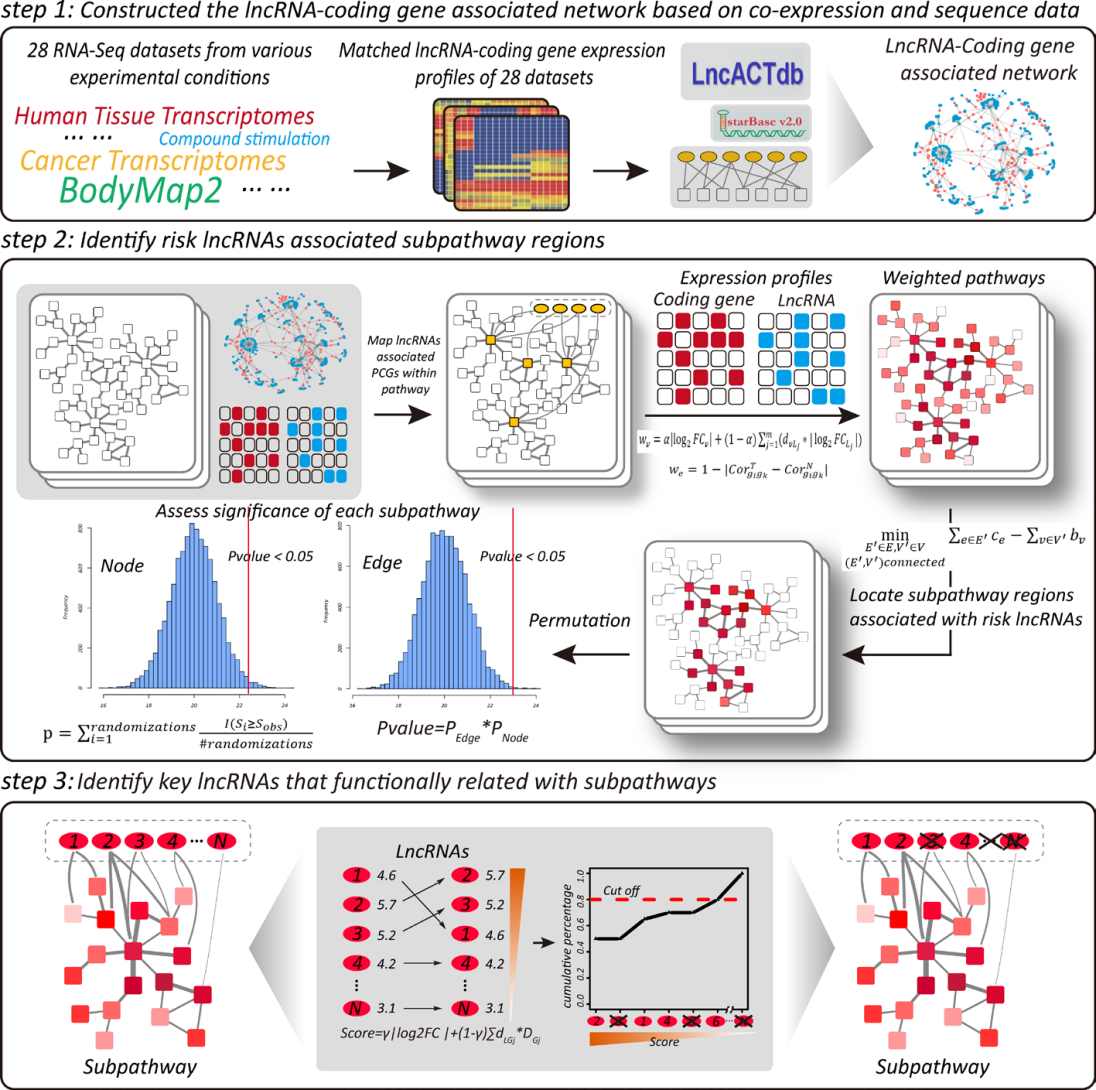
Schematic overview of LncSubpathway

LncSubpathway has been implemented as a freely-available web server (http://www.bio-bigdata.com/lncSubpathway/). A schematic overview of LncSubpathway is shown in Figure 7. A detailed description of method is provided in the following sections and in the Supplementary Text.

### Constructing the global lncRNA-protein coding gene association network

In this study, we constructed the global lncRNA-mRNA association network by integrating two distinct but complementary data sets: (1) the lncRNA-mRNA co-expression network; and (2) the lncRNA-mRNA ceRNA network.

We constructed a lncRNA-mRNA association network based on correlations between lncRNA and mRNA expression across 28 RNA-seq datasets that reflected different experimental conditions. Detailed processing for each RNA-seq dataset was conducted as follows: (i) individual lncRNAs or protein coding genes were used to construct the co-expression network only if the coefficient of variation for its expression across samples in the dataset was ≥ 0.3 and also ranked in the top 75 percentile of all coefficients. (ii) Pearson correlation coefficients (PCC) were calculated for each gene pair that met the above criteria based on the expression profiles. (iii) The statistical significance of each PCC value was determined using Fisher's asymptotic test to calculate *P*-values for each gene pair with the WGCAN R package [67]; these *P*-values were corrected using the Bonferroni adjustment for multiple tests. (iv) For each gene, co-expression pairs of it and other genes with adjusted *P* < 0.01 and PCC values ranked in the top or bottom 0.1% of all co-expression pairs were retained. Finally, only lncRNA-mRNA pairs for which the direction of the significant correlation (positive or negative) was consistent in at least three different datasets were included in the final co-expression network.

We then constructed a lncRNA-mRNA association network based on the ceRNA hypothesis. Briefly, lncRNA and mRNA pairs were retained for use in constructing the network only if they shared enough miRNAs. The details of this selection process were as follows: (i) We integrated experimentally validated mRNA-miRNA interactions from the TarBase [68], mirTarBase [69], mir2Disease [70], and miRecords (V4.0) [71] databases. (ii) lncRNA-miRNA interactions identified in our previously study [64], which examined lncRNA-associated competing triplets, were included. In addition, lncRNA and miRNA associations stored in the StarBase database [72] were also integrated. (iii) We constructed lncRNA-mRNA association relationships based on shared miRNAs. We identified protein coding genes as associated with a lncRNA when the lncRNA-mRNA pair simultaneously satisfied these two criteria: (1) the hypergeometric test for shared miRNAs was statistically significant (*P* < 0.05); (2) the Jaccard Coefficient of the shared miRNAs was in the top 20% of the overall mRNA list. The hypergeometric test formula was as follows:

$$P=1-\sum_{k=0}^{m}\frac{\left(\begin{array}{c} n\\ k \end{array}\right)\left(\begin{array}{c} N-n\\ M-k \end{array}\right)}{\left(\begin{array}{c} N\\ M \end{array}\right)}$$ (1)

Where *N* is all of miRNAs that interact with lncRNA/mRNA, *M* is the number of miRNAs that interact with the given mRNA, *n* is the number of miRNAs that interact with the given lncRNA, *m* is the number of miRNAs that interact with both the given lncRNA and mRNA.

We then integrated the lncRNA-protein coding gene co-expression network and the lncRNA-protein coding gene association network constructed based on ceRNA hypothesis. Ultimately, the global lncRNA-protein coding gene network included 6,037 lncRNAs, 8,967 PCGs, and 24,393 associations.

### Locating subpathways with risk lncRNA-associated transcriptional dysregulation

First, we converted KGML files containing protein-protein interaction and biochemical reaction information for a total of 281 pathways downloaded from the KEGG database in April 2015 into undirected graphs using our previously developed package [17]. Briefly, for each pathway, we extracted all genes identified as nodes in the corresponding graph. If a protein node in the KEGG graph interacted with another protein, an edge was used to connect the genes associated with one protein node to those associated with the other. For metabolic pathways, if a metabolite was both the product of the reaction involving one enzyme and the substrate of the reaction involving another enzyme in the pathway (i.e. two enzymes shared common metabolites), an edge was used to connect the two enzymes (genes). We thus used the original KEGG pathways to generate new graphs that maintained the original pathway topologies. Disease pathways and pathways for which structures could not be efficiently extracted were excluded. 214 pathways were retained for further analysis after screening.

Next, we calculated node weights and edge weights for each pathway based on the matched lncRNA-mRNA expression profiles, pathway topologies, and the global lncRNA-mRNA association network. Pathways that involved at least one gene regulated by lncRNAs of interest were assigned weights. Node (PCGs in given pathway) weights were assigned based on differential expression of each gene between the experimental condition and the corresponding controls, the differential expression of lncRNAs associated with the node, and the change in the correlation between the gene and lncRNAs. Specifically, for a given node (PCG) *v*, *L*1*,…,L*m represents each of the m lncRNAs that are associated with *v*, and the weight of node *v*, *b_v_*, is calculated using the following formula:

$$\;w_{v}=\alpha \left|\log_{2}FC_{v}\right|+(1-\alpha )\sum_{j=1}^{m}\left(d_{vL_{j}}*\left|\log_{2}FC_{L_{j}}\right|\right)$$ (2)

$$d_{vL_{j}}=1+\left|Cor_{vL_{j}}^{T}-Cor_{vL_{j}}^{N}\right|$$ (3)

Where *FC_v_* is the fold-change in expression for node *v*, *FC_Lj_* is the fold-change in expression of lncRNA *L*j; *Cor*_VLj_ and *Cor*_VLj_correspond to the Pearson Correlation Coefficient between gene *V* and lncRNA *L*j in tumor and normal states, respectively; and α is a constant value, which was 0.5 in this study. We then normalized the weight of each node within a given pathway as follows:

$$b_{v}=\beta *\left(w_{v}-w_{min}\right)/w_{max}$$ (4)

Where *w_min_* and *w_max_* are the minimum and maximum weight values, respectively, of nodes within a given pathway, and β is a constant value, which was 15 in this study. The edge weight for each pathway corresponds to the change in the interaction between connected gene pairs within the pathway. The edge weight for gene pair (*g*_i_, *g*_k_),*C_e_*, was calculated using the following formula:

$$w_{e}=1-\left|Cor_{g_{i}g_{k}}^{T}-Cor_{g_{i}g_{k}}^{N}\right|$$ (5)

$$c_{e}=\left(w_{e}-w_{emin}\right)/w_{max}$$ (6)

Where *w_emin_* and *w_emax_* correspond to the minimum and maximum weight values, respectively, of edges within a given pathway, and *Cor*_gigk_ and *Cor*_gigk_refer to the Pearson Correlation Coefficient between gene *g*i and gene *g*k in tumor and normal states, respectively.

Finally, we used the PCST algorithm [21–23] to locate subpathway regions containing the most dysregulated genes related to risk lncRNAs with connections that were substantially altered within the overall pathway graph G = (V, E) . Formally, the PCST algorithm identifies a connected subgraph *G' = (V', E)* that minimizes the following function:

$$\min\;{\;}_{\begin{array}{c} E^{\prime }\in E,V^{\prime }\in V\\ (E^{\prime },V^{\prime })\;connected \end{array}}\;\sum_{e\in E^{\prime }}c_{e}-\;\sum_{v\in V^{\prime }}b_{v}$$ (7)

We used the same solution to the PCST algorithm that was used in Bailly-Bechet *et* *al*. [21] to locate dysregulated subpathways that were functionally associated with risk lncRNAs.

### Evaluating the statistical significance of subpathways

For each subpathway, we defined the subpathway node (edge) weight, *S*_obs_ (*S*_obs_), as the mean value of all node (edge) weights which have not been normalized (i.e. *w_v_ (W_e_)*) within the subpathway. We performed 1000 randomizations on the node and edge set to evaluate the significance of each identified subpathway. For each node set, we randomly selected the same number of nodes contained in the subpathway from the background node set, which included all PCGs that were associated with at least one lncRNA of interest. For each edge set, we randomly selected the same number of edges contained in the subpathway from the background edge set, which included all edges from all pathways. Then, *S*_rand_ (*S*_rand_) was calculated as the mean value of the random node (edge) weights for each permutation. The *P*-value estimate for each subpathway was computed as follows:

$$p=\sum_{i=1}^{randomizations}\frac{I(S_{i}>S_{obs})}{\#randomizations}$$ (8)

where *S_i_* represents *S*_rand_ and *I(S_i_ > S_obs_* and is an indicator function which equals 1 when the i^th^ random node (edge) weight, *S_i_*, is equal or greater than the observed value (*S_obs_*); otherwise, it equals 0.

Finally, we joined the subpathway *P*-values at both the node and edge levels to evaluate the significance of individual subpathways using the formula *p_ji_ = p_vi_ p_ei_* where *p_ji_, p_vi_*, and *p_ei_* represent the joint *P*-value and the *P*-values obtained at the node and edge levels for subpathway *i*, respectively.

### Identifying key lncRNAs

We identified key lncRNAs that were associated with transcriptional dysregulation for each subpathway by considering lncRNA dysregulation, alterations in the correlation between a lncRNA and the PCGs with which it interacted, and the topological position of these PCGs within the subpathway. Briefly, we aimed to identify a minimum core lncRNA set that was associated with most of the PCGs within the subpathways that exhibited transcriptional dysregulation. To do this, we first ranked the lncRNAs associated with a given subpathway according to the importance score (IS) of the lncRNA, which was calculated as follows:

$$IS=\gamma \left|\log_{2}FC\right|+(1-\gamma )\sum_{j=1}^{n}d_{LG_{j}}*D_{G_{j}}$$ (9)

Where *FC* denotes the fold-change value for lncRNA *L*, *G*_1_*,…,G*_n_ represents the *n* PCGs that interact with *L* within the subpathway, *D_gj_* denotes the degree (a topology measurement) of gene *G*j within the subpathway, and *d_LGj_* represents the change in the correlation between *L* and *G*j, which is calculated as in equation (3). γ is 0.3 in this study.

We then identified the key lncRNAs as follows:

(i) After designating the lncRNA with the highest IS value the core lncRNA, the proportion (q) of genes with which it was associated within the subpathway was calculated.

(ii) If the proportion of genes associated with the above lncRNA(s) was less than a given cutoff pert, then the next lncRNA in the ranked list was considered for addition to the core lncRNA set and the new proportion parameter q', which indicated the proportion of genes associated with the new core lncRNA set, was calculated. If q' > q, the relevant lncRNA was added to the core lncRNA set; otherwise, it was removed.

(iii) The above step was repeated until q ≥ pert ; lncRNAs included in the core lncRNA set were identified as key lncRNAs. In this study, pert was set at 0.8.

### Simulation designs

We performed three simulation experiments to evaluate the LncSubpathway method. Briefly, datasets with 150 genes and 50 lncRNAs each were generated from two genetic systems (i.e. two pathway networks). The two pathway network models are the linear pathway, with 20 genes that were connected in a linear fashion (Supplementary Figure 6A), and the ERBB signaling pathway, with genes that interacted with each other according to the ERBB signaling pathway in the KEGG database (Supplementary Figure 6B). These two pathways were assumed to have no interactions with each other. The lncRNA/mRNA expression profiles were generated using a multivariate normal distribution model, and the lncRNA-mRNA association network was generated using a random network model (details see Supplementary Text).

The first simulation explored the characteristics of LncSubpathway by varying the following parameters: sample size, differentiality of lncRNAs/PCGs, differentiality of interactions between PCG-PCG within subpathways, and associations between pathway PCGs and lncRNAs. This simulation experiment was conducted to demonstrate that the subpathway node (edge) weights increased, and the corresponding *P*-values decreased, as the differentiality of pathway-associated nodes (edges) increased. To that end, we generated simulated lncRNA and mRNA profiles by varying parameters *n, e and p* as follows: *n*, which controls to the fold-change of lncRNAs/PCGs, was varied from 2.0 to 7.0 in increments of 0.5; *e*, which controls the extent to which interactions changed, was varied from 0.1 to 0.9 in increments of 0.1; and *p*, which determines the proportion of pathway-associated lncRNAs/PCGs or associations that changed, was varied from 0.1 to 0.9 in increments of 0.2 (see the Supplementary Text). The sample size, N, was set at 250, 300, or 500. Each unique combination of these parameters (e.g. *n* = 2.0, *p* = 0.1, and *N* = 250) was defined as a single simulation case. For each simulation case, simulated datasets were generated and analysis using LncSubpathway was repeated 100 times. A detailed description of the simulation experiments is provided in the Supplementary Text.

The second simulation evaluated the false positive rates of LncSubpathway using two other simulation strategies from Choi *et al*. [73] and Goel *et al*. [74] to generate a simulated dataset. Statistically significant simulation cases obtained when the method is applied to a dataset with *p*=0 (i.e. no changes between two sample groups) were designated false positives. In the dataset generated using both strategies, the mean expression of PCGs/lncRNAs was equal (μ_1_ = μ_2_) and the correlations among lncRNAs/PCGs were equal (∑1 = ∑2) in the two sample groups. Simulation dataset generation was repeated 100 times under each simulation parameter condition for both strategies. False positive rates were estimated by observing the proportion of replicates with a *P* < 0.01. The two strategies differed in the parameter settings used to generate the simulated datasets; a detailed description of the two simulation scenarios is provided in the Supplementary Text.

The third simulation evaluated whether LncSubpathway accurately located dysregulated subpathway regions that were associated with lncRNAs of interest. We assumed that one subpathway region in the linear pathway and three subpathway regions in the ERBB pathway were dysregulated (Supplementary Figure 1). Then, we generated simulation datasets that satisfied the requirement for differential expression in the focal subpathway regions. Simulated datasets were generated independently 100 times each using node (lncRNA/PCG) fold-changes of 1.15, 1.5, 2.0, 2.5, 3.0, 3.5, 4.0, 4.5, 5.0, 5.5, 6.0, 6.5, or 7.0, interaction changes of 0.1, 0.2, 0.3, 0.4, 0.5, 0.6, 0.7, 0.8, or 0.9, and sample sizes of 250, 300, or 500 as input conditions (see the Supplementary Text for details). We then calculated the ratio of genes involved in the given subpathway region from Supplementary Figure 1 that was recovered in each replicate. The average values of repeats for each simulation condition were used to evaluate the accuracy of LncSubpathway in locating dysregulated subpathway regions. A detailed description of this method is provided in the Supplementary Text.

## SUPPLEMENTARY MATERIALS FIGURES AND TABLES


