# Supplementary material for: LncSubpathway: a novel approach for identifying dysfunctional subpathways associated with risk lncRNAs by integrating lncRNA and mRNA expression profiles and pathway topologies
Source: Oncotarget. 2017 Feb 1;8(9):15453–69. doi: 10.18632/oncotarget.14973 (PMC5362499; doi:10.18632/oncotarget.14973)
Supplement: Supplementary file 1 [file oncotarget-08-15453-s001.pdf]

LncSubpathway: a novel approach for identifying dysfunctional subpathways associated with risk lncRNAs by integrating lncRNA and mRNA expression profiles and pathway topologies

Supplementary Materials

SUPPLEMENTARY TEXT

Simulation experiments

In this section, we performed three simulation experiments to evaluate LncSubpathway method. The simulation datasets were generated from two genetic systems each with 150 genes and 50 lncRNAs.

Simulated two pathway structure models

In the two genetic systems, one had a linear pathway with 20 genes (nodes) which are interacted as a linear fashion (Supplemantary Figure 6A); the other had an ERBB signaling pathway with 43 genes (nodes) which are interacted as the same manner in ERBB signaling pathway of KEGG database (Supplemantary Figure 6B). For each simulation case, we simulated the lncRNA-PCG interaction network by generating random network and simulated the matched lncRNA, mRNA expression profile by using the multivariate normal distribution model. And then applied LncSubpathway to locate subpathways associated with dysregulated lncRNAs. Details of each simulation experiment were as follows.

Generation of lncRNA-PCG interaction network

In each of these two genetic system, we assumed that there have 150 background genes and 50 background lncRNAs. To generate the simulated lncRNA-PCG interaction network, we firstly defined two parameter vectors: (1) the degree of PCG nodes in the generated network varying from 1 to 5; (2) weight vector (0.6,0.25,0.1,0.025,0.025), which determined the corresponding probability of degree (1,2,3,4,5) for each PCG nodes. In detail, for each of these 150 genes in the given genetic system, we randomly defined the degree of a given gene from 1 to 5 according to the probability vector (0.6,0.25,0.1,0.025,0.025). Then, we randomly selected the number of lncRNAs from background according to the degree determined in the above and connected these lncRNAs to this gene. In this study, for each simulation case and repeats, the lncRNA-PCG interaction network were regenerate.

Generation of background PCG-PCG interaction edges

In order to evaluate the significance P-value of pathway in each simulation, we also need a background edge set. To do this, we firstly removed genes in the focused pathway (Linear or ERBB) from the 150 background genes and thus the number of retained genes is v. Then, we randomly selected 2*v edges from the all possible gene pairs constructed by the v retained genes as background PCG-PCG interaction edges to evaluate the significance of located subpathways.

Generation of matched lncRNA/mRNA expression profiles

The expression profiles of two sample groups of equal size N were simulated from P-dimensional normal distributions N(μ1,Σ 1) and N(μ2,Σ 2)representing two biological conditions (e.g. Normal versus Tumor). In this simulation, P is the total number of background genes and lncRNAs. Mean vector μ1 were generated as uniform and random variables in interval (0.1,10) and Σ 1 is a unit matrix. The definition of parameter μ2 and Σ 2 were different according to different purpose of simulation experiments. The detail value of μ1,Σ 1,μ2 and Σ 2 were defined as follows. Sample size N is chosen among (250,300,500) in each simulation experiments.

Simulation I is to characterize LncSubpathway with varying parameters such as sample size, differentiality of lncRNAs/PCGs and differentiality of interactions between PCG-PCG within pathway and interactions between lncRNAs and PCGs of pathway. The aim of this simulation experiment is to show that the node (edge) weight of pathway increases and corresponding significance *P*-value decreases as the increase of node (edge)differentiality associated with the pathway.

Node change

To explore how the change of lncRNAs/PCGs impact the weight and significance *P*-value of located subpathways, we generated the matched lncRNA/mRNA expression profiles as follows. Firstly, we defined the parameters used in this section including n andp; where n is the fold change of differential PCGs or lncRNAs, p is the proportion of

differential PCGs or lncRNAs.For the P-dimensional normal distributions N(μ1,Σ 1) and N(μ2,Σ 2), mean vector μ1 were generated as uniform and random variables in interval (0.1,10) and Σ 1 is a unit matrix.Σ 2 is defined to equal Σ1.The elements of mean vector μ2 is defined as follows:

$$\mu_i^2 = \begin{cases} \mu_i^1, & if\ i \in S_{nd}^{PCG} \\ n * \mu_i^1, & if\ i \in S_d^{PCG}\ or\ i \in S_d^{Lnc} \\ 2 * \mu_i^1, & if\ i \in S_{nd}^{Lnc} \end{cases} \quad (1)$$

í is a PCG or lncRNA, μ_i^1 is the value of i in mean vector μ1, S_{nd}^{PCG} is the non-differential PCG set, S_{nd}^{Lnc} is the non-differential lncRNA set, S_d^{PCG} is the differential PCG set and S_d^{Lnc} is the differential lncRNA set.

The simulation experiments were performed on changes of PCGs within pathway, changes of lncRNAs and changes of both PCGs and lncRNAs, respectively. For simulation experiments that focused on changes of PCGs within pathway, we randomly selectedp proportion of PCGs within the focused pathway (Linear or ERBB) as differential PCGs. For simulation experiments that focused on changes of lncRNAs, we randomly selectedp proportion of lncRNAs that interacted with PCGs within the focused pathway (Linear or ERBB) as differential lncRNAs. For simulation experiments that focused on changes of both PCGs and lncRNAs, we randomly selectedp proportion of PCGs within the focused pathway (Linear or ERBB) and p proportion of lncRNAs that interacted with PCGs within the focused pathway as differential nodes.The parameter n controls the strength of change, which varied from 2 to 7 with 0.5 interval. The proportion of differential interactions p also varied from 0.1 to 0.9 with 0.1 interval.Onesimulation case refers as one combination of each parameter. For example, simulation case ($n = 2.0$, $p = 0.1$ and $N = 250$) represent that 10% percentage of PCGs in the pathway or lncRNAs that interacted with the pathway PCGs were 2 Fold-changed and other PCGs (lncRNAs) in the genetic system were not changed, the generated expression profiles with 250 samples. For each simulation case, the LncSubpathway method was repeated 100 times.

More specifically, in order to highlight the impact of change lncRNAs on the weight and significance *P*-value of located subpathway, inthesimulation experiments that focused on changes of lncRNAs, we modified the generation of lncRNA-PCG association network step: (i) Firstly, we randomly selected 15 lncRNAs from the background lncRNAs; (ii) we constructed the lncRNA-PCG associations between these 15 lncRNAs and PCGs in focused pathway (Linear or ERBB) based on the same strategy used in above; (iii) associations between the other lncRNAs and PCGs not in the pathway were connected by the above strategy; (iv) the above two lncRNA-PCG interaction sets were connected as the final generated lncRNA-PCG association network.

Edge change

To explore how the change of interactions between PCGs within pathway and interactions between lncRNAs and their regulated pathway PCGs impact the weight and significance *P*-value of located subpathways, we performed the following simulations.

In the simulation experiment, the "*Generation of matched lncRNA/mRNA expression profiles*" step, the parameter setting were as follows:

Firstly, we defined the parameters used in this section including e, p and N; where e is the change value of differential interactions, p is the proportion of differential interactions and N is the number of samples in the simulation profiles.Two sample groups of equal size N were simulated from P-dimensional normal distributions N(μ1, Σ1) and N(μ2, Σ2)representing two biological conditions (e.g. Normal versus Tumor).In this simulation, P is the total number of background genes and lncRNAs. Mean vector μ1 were generated as uniform and random variables in interval (0.1,10) and values in μ2 is 2 fold of the corresponding value in μ1. The matrixΣ 1 is defined as a unit matrix. The elements of matrix Σ 2 is defined as follows:

$$\sigma_{ij} = \sigma_{ji} = \begin{cases} 1, & if\ i = j \\ e, & if\ the\ correlation\ between\ i\ and\ j\ is\ differential \\ 0, & otherwise \end{cases} \quad (2)$$

For matrix Σ2, column sumsare computed from the absolute values of matrix entries, and thecorresponding diagonal element is set to the sum plus a smallconstant (0.0001). Then,diagonal elements of matrix Σ1 were assigned the same as that of Σ2.

The simulation experiments were performed on changes of interactions between PCGs within pathway, changes of interactions between lncRNAs and pathway PCGs and changes of both of these two interaction types, respectively. Forsimulation experiments that focused on changes of interactions between PCGs within pathway, we randomly selectedp proportion of edges within the focused pathway (Linear or ERBB) as differential interactions. For simulation experiments that focused on changes of interactions between lncRNAs and pathway PCGs, we randomly selectedp proportion of interactions between lncRNAs and the focused pathway (Linear or ERBB) PCGs as differential interactions.For simulation experiments that focused on changes of both of these two interaction types, we randomly selectedp proportion of edges within the focused pathway (Linear or ERBB) PCGs and p proportion of interactions between lncRNAs and the focused pathway PCGs as differential interactions. The parameter e controls the strength of correlation between PCGs within pathway or the strength of correlation between lncRNAs and their regulated

pathway PCGs, which varied from 0.1 to 0.9 with 0.1 interval. The proportion of differential interactions p also varied from 0.1 to 0.9 with 0.1 interval.

Simulation II is to evaluate the false positive rate of LncSubpathway. We evaluated the false positive rates of LncSubpathway using two simulation strategies to generate the simulated expression dataset, which were used in the study of Choi et al. [1] and the study of Goel et al. [2] to evaluate the false positive rates of their methods, respectively. The detailed description of these two methods were as follows.

The first strategy for generation of simulated expression profile is similar with the study of Choi et al.. The expression profiles of two sample groups of equal size N were simulated from P-dimensional normal distributions as described in the 'Generation of matched lncRNA/mRNA expression profiles' section. In detail, mean vector μ1 were generated as uniform and random variables in interval (0.1,10). For matrix Σ1,off-diagonal positions in the upper triangular portion of the matrix are filled in with random draws from a uniform distribution between −1 and 1. The lower triangularportion is filled in to create a symmetric matrix.Column sums are computed from the absolute values of matrix entries, and thecorresponding diagonal element is set to the sum plus a smallconstant. In this study, the constant is set as 0.0001.Σ2 is defined to equal Σ1 and μ2 is defined to equal μ1.The sample size N was chose among (250,300,500). For each sample size, the simulation experiment was repeated 1000 times for each model pathway (Linear or ERBB). False positive rates were estimated by the observed proportion ofreplicates with a *P*-value < 0.01.

The second strategy for generation of simulated expression profile is similar with the study of Goel et al.. In detail, mean vector μ1 were generated as uniform and random variables in interval (0,10). For matrix Σ1, the 200 diagonal elements were generated as uniform and random variables in interval (1, 10).The off-diagonal elements of the matrix werevaried with a correlation (r) of 0.1, 0.2, 0.3, 0.4, 0.5, 0.6, 0.7, 0.8 and 0.9.The sample size N was also chosen among (250,300,500). The simulation dataset were generated 100 replicates under each condition (e.g. *N* = 250, r = 0.1).False positive rates were estimated by the observed proportion ofreplicates with a *P*-value < 0.01.

Simulation III aims to evaluate whether LncSubpathway can accurately locate dysregulated subpathway regions that mediated by interesting lncRNAs. We firstly assumed one subpathway region in Linear pathway (Supplementary Figure 1A) and three subpathway regions in ERBB pathway (Supplementary Figure 1B) was dysregulated and other regions within pathway was not altered. Three regions in ERBB pathway was analyzed separately. Then, the simulation experiments that respectively focused on these four subpathway regions were performed. To illustrate how the simulated dataset generated, we take the ERBB subpathway region 1 shown in Supplementary Figure 1B as example.The simulated dataset make that PCGs within the ERBB subpathway region 1 and lncRNAs regulated these PCGs were all with fold-change n and other lncRNAs and PCGs were have equal expression mean value under two conditions. At the same time, correlation of all edges within the subpathway region and interactions between PCGs in subpathway and lncRNAs were differential with e, while other interactions and edges were not. The generation of simulation expression profiles that satisfy the above situations is as follows. Mean vector μ1 were generated as uniform and random variables in interval (0.1,10) and Σ1 is a unit matrix. The elements of mean vector μ2 is defined as follows:

$$\mu_i^2 = \begin{cases} n * \mu_i^1,\ if\ i \in S_d^{PCG}\ or\ i \in S_d^{Lnc} \\ \mu_i^1,\ otherwise \end{cases} \quad (3)$$

Where i is a PCG or lncRNA, μ_i^1 is the value of i in mean vector μ1, S_d^{PCG} is the differential PCG set and S_d^{Lnc} is the differential lncRNA set. The elements of matrix Σ 2 is defined as equation (2). For matrix Σ2, column sumsare computed from the absolute values of matrix entries, and thecorresponding diagonal element is set to the sum plus a smallconstant (0.0001). Then, diagonal elements of matrix Σ1 were assigned the same as that of Σ2.Sample size N were chosen among (250, 300,500). Parameter nvaried among 1.15,1.5,2.0,2.5,3.0,3.5,4.0,4.5,5.0,5.5,6.0,6.5 and 7.0; while e varied 0.1,0.2,0.3,0.4,0.5,0.6,0.7,0.8 and 0.9. The simulation dataset were generated 100 replicates under each condition (e.g. *N* = 250, *n* = 2 and e = 0.1).For each repeat, we calculated the ratio of genes involved in the ERBB subpathway region 1 that recovered in the located subpathway. Average value for repeats under each simulation case were used to evaluate the accuracy of LncSubpathway to locate dysregulated subpathway regions.

REFERENCES

1. Choi Y, Kendziorski C. Statistical methods for gene set co-expression analysis. Bioinformatics. 2009; 25:2780–2786.
2. Goel G, Conway KL, Jaeger M, Netea MG, Xavier RJ. Multivariate inference of pathway activity in host immunity and response to therapeutics. Nucleic acids research. 2014; 42:10288–10306.
3. Levesque N, Christensen KE, Van Der Kraak L, Best AF, Deng L, Caldwell D, MacFarlane AJ, Beauchemin N, Rozen R. Murine MTHFD1-synthetase deficiency, a model for the human MTHFD1 R653Q polymorphism, decreases growth of colorectal tumors. Molecular carcinogenesis 2016.
4. MacFarlane AJ, Perry CA, McEntee MF, Lin DM, Stover PJ. Mthfd1 is a modifier of chemically induced intestinal carcinogenesis. Carcinogenesis. 2011; 32:427–433.
5. Tolstikov V, Nikolayev A, Dong S, Zhao G, Kuo MS. Metabolomics analysis of metabolic effects of nicotinamide

phosphoribosyltransferase (NAMPT) inhibition on human cancer cells. PLoS One. 2014; 9:e114019.

6. Sakai E, Fukuyo M, Matsusaka K, Ohata K, Doi N, Takane K, Matsuhashi N, Fukushima J, Nakajima A, Kaneda A. TP53 mutation at early stage of colorectal cancer progression from two types of laterally spreading tumors. Cancer science. 2016; 107:820–827.

7. Li Y, Sun Y, Fan L, Zhang F, Meng J, Han J, Guo X, Zhang D, Zhang R, Yue Z, Mei Q. Paris saponin VII inhibits growth of colorectal cancer cells through Ras signaling pathway. Biochemical pharmacology. 2014; 88:150–157.

8. Sun D, Yu F, Ma Y, Zhao R, Chen X, Zhu J, Zhang CY, Chen J, Zhang J. MicroRNA-31 activates the RAS pathway and functions as an oncogenic MicroRNA in human colorectal cancer by repressing RAS p21 GTPase activating protein 1 (RASA1). The Journal of biological chemistry. 2013; 288:9508–9518.

9. Khanna A, Lotfi P, Chavan AJ, Montano NM, Bolourani P, Weeks G, Shen Z, Briggs SP, Pots H, Van Haastert PJ, Kortholt A, Charest PG. The small GTPases Ras and Rap1 bind to and control TORC2 activity. Scientific reports. 2016; 6:25823.

10. Lee JW, Ryu YK, Ji YH, Kang JH, Moon EY. Hypoxia/reoxygenation-experienced cancer cell migration and metastasis are regulated by Rap1- and Rac1-GTPase activation via the expression of thymosin beta-4. Oncotarget. 2015; 6:9820–9833. doi: 10.18632/oncotarget.3218.

11. Temraz S, Mukherji D, Shamseddine A. Dual Inhibition of MEK and PI3K Pathway in KRAS and BRAF Mutated Colorectal Cancers. International journal of molecular sciences. 2015; 16:22976–22988.

12. Raynal NJ, Lee JT, Wang Y, Beaudry A, Madireddi P, Garriga J, Malouf GG, Dumont S, Dettman EJ, Gharibyan V, Ahmed S, Chung W, Childers WE, et al. Targeting Calcium Signaling Induces Epigenetic Reactivation of Tumor Suppressor Genes in Cancer. Cancer research. 2016; 76:1494–1505.

13. Tolba MF, Abdel-Rahman SZ. Pterostilbine, an active component of blueberries, sensitizes colon cancer cells to 5-fluorouracil cytotoxicity. Scientific reports. 2015; 5:15239.

14. Wiegering A, Uthe FW, Jamieson T, Ruoss Y, Huttenrauch M, Kuspert M, Pfann C, Nixon C, Herold S, Walz S, Taranets L, Germer CT, Rosenwald A, et al. Targeting Translation Initiation Bypasses Signaling Crosstalk Mechanisms That Maintain High MYC Levels in Colorectal Cancer. Cancer discovery. 2015; 5:768–781.

15. Leon J, Casado J, Jimenez Ruiz SM, Zurita MS, Gonzalez-Puga C, Rejon JD, Gila A, Munoz de Rueda P, Pavon EJ, Reiter RJ, Ruiz-Extremera A, Salmeron J. Melatonin reduces endothelin-1 expression and secretion in colon cancer cells through the inactivation of FoxO-1 and NF-kappabeta. Journal of pineal research. 2014; 56:415–426.

16. Coomans de Brachene A, Demoulin JB. FOXO transcription factors in cancer development and therapy. Cellular and molecular life sciences. 2016; 73:1159–1172.

17. He Q, Xu Q, Wu W, Chen L, Sun W, Ying J. Comparison of KRAS and PIK3CA gene status between primary tumors and paired metastases in colorectal cancer. OncoTargets and therapy. 2016; 9:2329–2335.

18. Jung SK, Jeong CH. Dehydroglyasperin D Inhibits the Proliferation of HT-29 Human Colorectal Cancer Cells Through Direct Interaction With Phosphatidylinositol 3-kinase. Journal of cancer prevention. 2016; 21:26–31.

19. Abruzzo A, Zuccheri G, Belluti F, Provenzano S, Verardi L, Bigucci F, Cerchiara T, Luppi B, Calonghi N. Chitosan nanoparticles for lipophilic anticancer drug delivery: Development, characterization and *in vitro* studies on HT29 cancer cells. Colloids and surfaces B, Biointerfaces. 2016; 145:362–372.

20. Tariq K, Ghias K. Colorectal cancer carcinogenesis: a review of mechanisms. Cancer biology & medicine. 2016; 13:120–135.

21. Wanzel M, Vischedyk JB, Gittler MP, Gremke N, Seiz JR, Hefter M, Noack M, Savai R, Mernberger M, Charles JP, Schneikert J, Bretz AC, Nist A, et al. CRISPR-Cas9-based target validation for p53-reactivating model compounds. Nature chemical biology. 2016; 12:22–28.

22. Kramer HB, Lai CF, Patel H, Periyasamy M, Lin ML, Feller SM, Fuller-Pace FV, Meek DW, Ali S, Buluwela L. LRH-1 drives colon cancer cell growth by repressing the expression of the CDKN1A gene in a p53-dependent manner. Nucleic acids research. 2016; 44:582–594.

23. Liu S, Tackmann NR, Yang J, Zhang Y. Disruption of the RP-MDM2-p53 pathway accelerates APC loss-induced colorectal tumorigenesis. Oncogene. 2016.

24. Lee MH, Hong SH, Park C, Kim GY, Leem SH, ChoiSH, Keum YS, Hyun JW, Kwon TK, Hong SH, Choi YH. Hwang-Heuk-San induces apoptosis in HCT116 human colorectal cancer cells through the ROS-mediated activation of caspases and the inactivation of the PI3K/Akt signaling pathway. Oncology reports. 2016; 36:205–214.

25. Shao Z, Cai Y, Xu L, Yao X, Shi J, Zhang F, Luo Y, Zheng K, Liu J, Deng F, Li R, Zhang L, Wang H, et al. Loss of the 14-3-3sigma is essential for LASP1-mediated colorectal cancer progression via activating PI3K/AKT signaling pathway. Scientific reports. 2016; 6:25631.

26. Sun Y, Tian H, Wang L. Effects of PTEN on the proliferation and apoptosis of colorectal cancer cells via the phosphoinositol-3-kinase/Akt pathway. Oncology reports. 2015; 33:1828–1836.

27. Bravou V, Antonacopoulou A, Papanikolaou S, Nikou S, Lilis I, Giannopoulou E, Kalofonos HP. Focal Adhesion Proteins alpha- and beta-Parvin are Overexpressed in Human Colorectal Cancer and Correlate with Tumor Progression. Cancer investigation. 2015; 33:387–397.

28. Fukumoto M, Kurisu S, Yamada T, Takenawa T. alpha-Actinin-4 enhances colorectal cancer cell invasion by suppressing focal adhesion maturation. PloS one. 2015; 10:e0120616.

29. Zhao J, Bulek K, Gulen MF, Zepp JA, Karagkounis G, Martin BN, Zhou H, Yu M, Liu X, Huang E, Fox PL, Kalady MF, Markowitz SD, et al. Human Colon Tumors

Express a Dominant-Negative Form of SIGIRR That Promotes Inflammation and Colitis-Associated Colon Cancer in Mice. Gastroenterology. 2015; 149:1860–1871 e1868.

30. Wang H, Flannery SM, Dickhofer S, Huhn S, George J, Kubarenko AV, Lascorz J, Bevier M, Willemsen J, Pichulik T, Schafmayer C, Binder M, Manoury B, et al. A coding IRAK2 protein variant compromises Toll-like receptor (TLR) signaling and is associated with colorectal cancer survival. The Journal of biological chemistry 2014, 289:23123–23131.

31. Wang Z, Tian A, Benchabane H, Tacchelly-Benites O, Yang E, Nojima H, Ahmed Y. The ADP-ribose polymerase Tankyrase regulates adult intestinal stem cell proliferation during homeostasis in Drosophila. Development. 2016; 143:1710–1720.

32. Tian A, Benchabane H, Wang Z, Ahmed Y. Regulation of Stem Cell Proliferation and Cell Fate Specification by Wingless/Wnt Signaling Gradients Enriched at Adult Intestinal Compartment Boundaries. PLoS genetics. 2016; 12:e1005822.

33. Slattery ML, Lundgreen A, Kadlubar SA, Bondurant KL, Wolff RK. JAK/STAT/SOCS-signaling pathway and colon and rectal cancer. Molecular carcinogenesis. 2013; 52:155–166.

34. Harvey KF, Zhang X, Thomas DM. The Hippo pathway and human cancer. Nature reviews Cancer. 2013; 13:246–257.

35. Zhang K, Hu Z, Qi H, Shi Z, Chang Y, Yao Q, Cui H, Zheng L, Han Y, Han X, Zhang Z, Chen T, Hong W. G-protein-coupled receptors mediate omega-3 PUFAs-inhibited colorectal cancer by activating the Hippo pathway. Oncotarget. 2016; 7:58315–58330. doi: 10.18632/oncotarget.11089.

36. Zhang L, Castanaro C, Luan B, Yang K, Fan L, Fairhurst JL, Rafique A, Potocky TB, Shan J, Delfino FJ, Shi E, Huang T, Martin JH, et al. ERBB3/HER2 signaling promotes resistance to EGFR blockade in head and neck and colorectal cancer models. Molecular cancer therapeutics. 2014; 13:1345–1355.

37. Yao YL, Shao J, Zhang C, Wu JH, Zhang QH, Wang JJ, Zhu W. Proliferation of colorectal cancer is promoted by two signaling transduction expression patterns: ErbB2/ErbB3/AKT and MET/ErbB3/MAPK. PloS one. 2013; 8:e78086.

38. Abe N, Hou DX, Munemasa S, Murata Y, Nakamura Y. Nuclear factor-kappaB sensitizes to benzyl isothiocyanate-induced antiproliferation in p53-deficient colorectal cancer cells. Cell death & disease. 2014; 5:e1534.

39. Wang Y, Ren F, Wang Y, Feng Y, Wang D, Jia B, Qiu Y, Wang S, Yu J, Sung JJ, Xu J, Zeps N, Chang Z. CHIP/Stub1 functions as a tumor suppressor and represses NF-kappaB-mediated signaling in colorectal cancer. Carcinogenesis. 2014; 35:983–991.

40. Hu B, Elinav E, Huber S, Strowig T, Hao L, Hafemann A, Jin C, Wunderlich C, Wunderlich T, Eisenbarth SC, Flavell RA. Microbiota-induced activation of epithelial IL-6 signaling links inflammasome-driven inflammation with transmissible cancer. Proceedings of the National Academy of Sciences of the United States of America. 2013; 110:9862–9867.

41. Zaki MH, Vogel P, Malireddi RK, Body-Malapel M, Anand PK, Bertin J, Green DR, Lamkanfi M, Kanneganti TD. The NOD-like receptor NLRP12 attenuates colon inflammation and tumorigenesis. Cancer cell. 2011; 20:649–660.

42. Neradugomma NK, Subramaniam D, Tawfik OW, Goffin V, Kumar TR, Jensen RA, Anant S. Prolactin signaling enhances colon cancer stemness by modulating Notch signaling in a Jak2-STAT3/ERK manner. Carcinogenesis. 2014; 35:795–806.

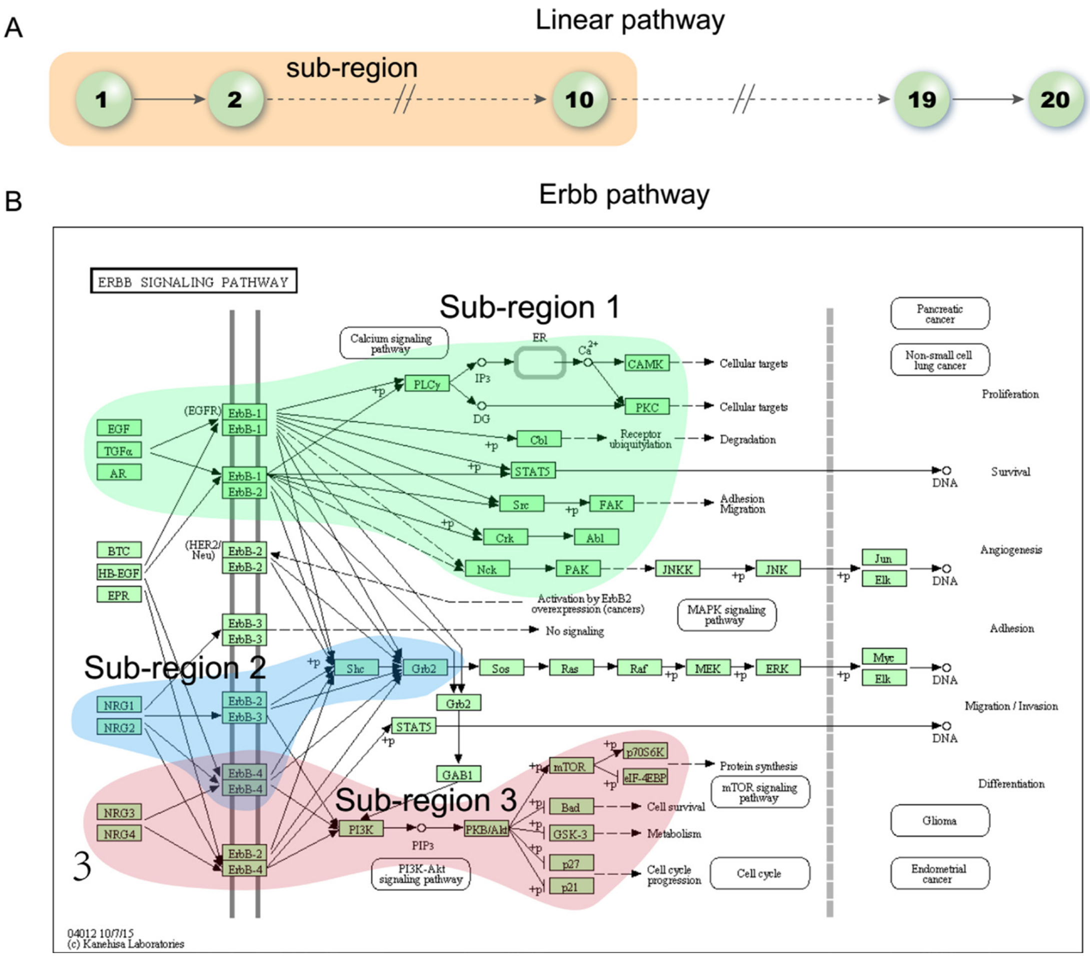

Supplementary Figure 1: Predefined dysregulated subpathway regions for evaluating the effectiveness of LncSubpathway based on simulation datasets. (**A**) The shaded region is the predefined dysregulated subpathway region in the linear pathway structure model. (**B**) The three shaded regions are the predefined dysregulated subpathway regions in the ERBB pathway structure model.

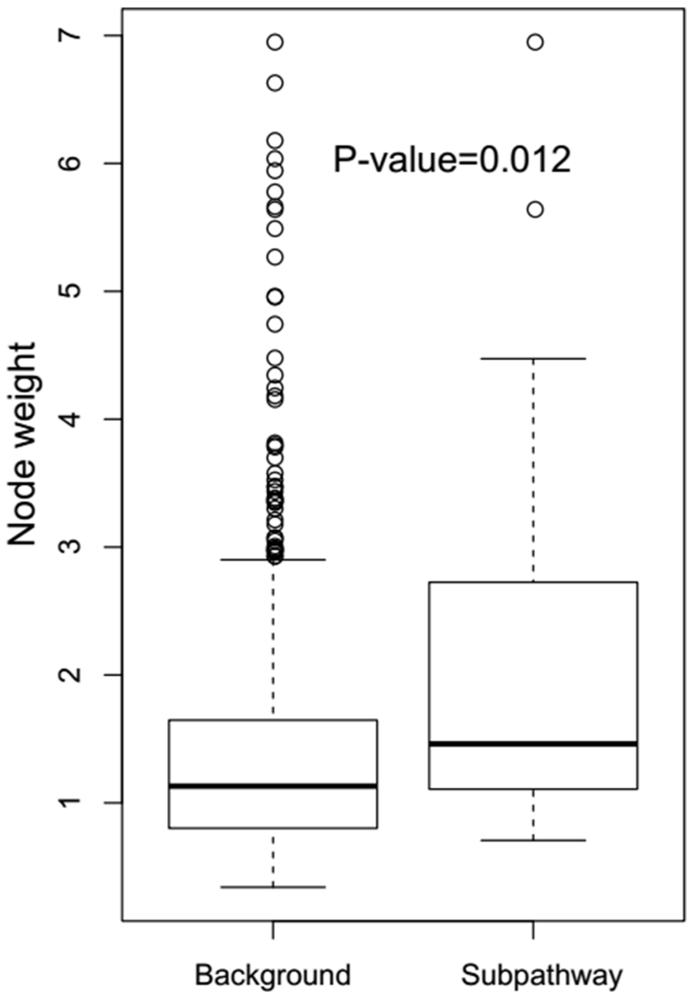

Supplementary Figure 2: Node weights within the FOXO subpathway region were higher than the background PCGs.

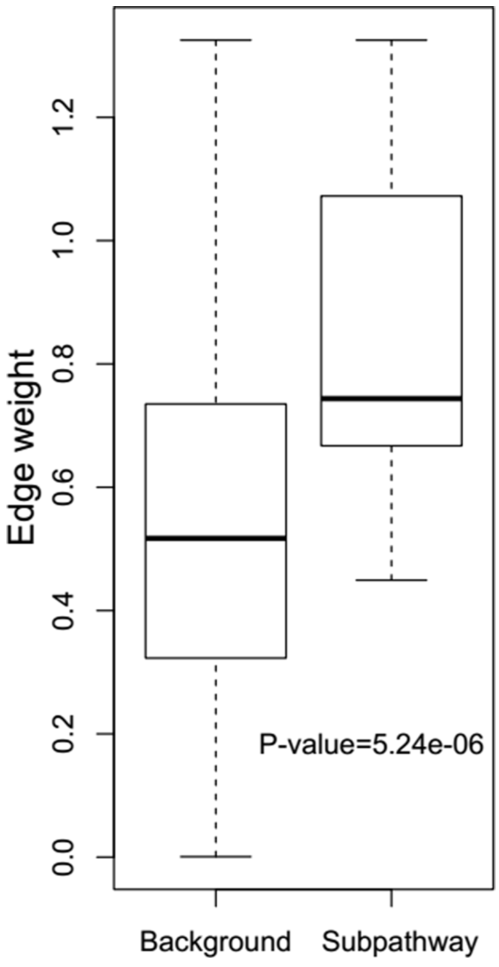

Supplementary Figure 3: The degree of change for correlations (edge weights) within the purine subpathway region was higher than the background.

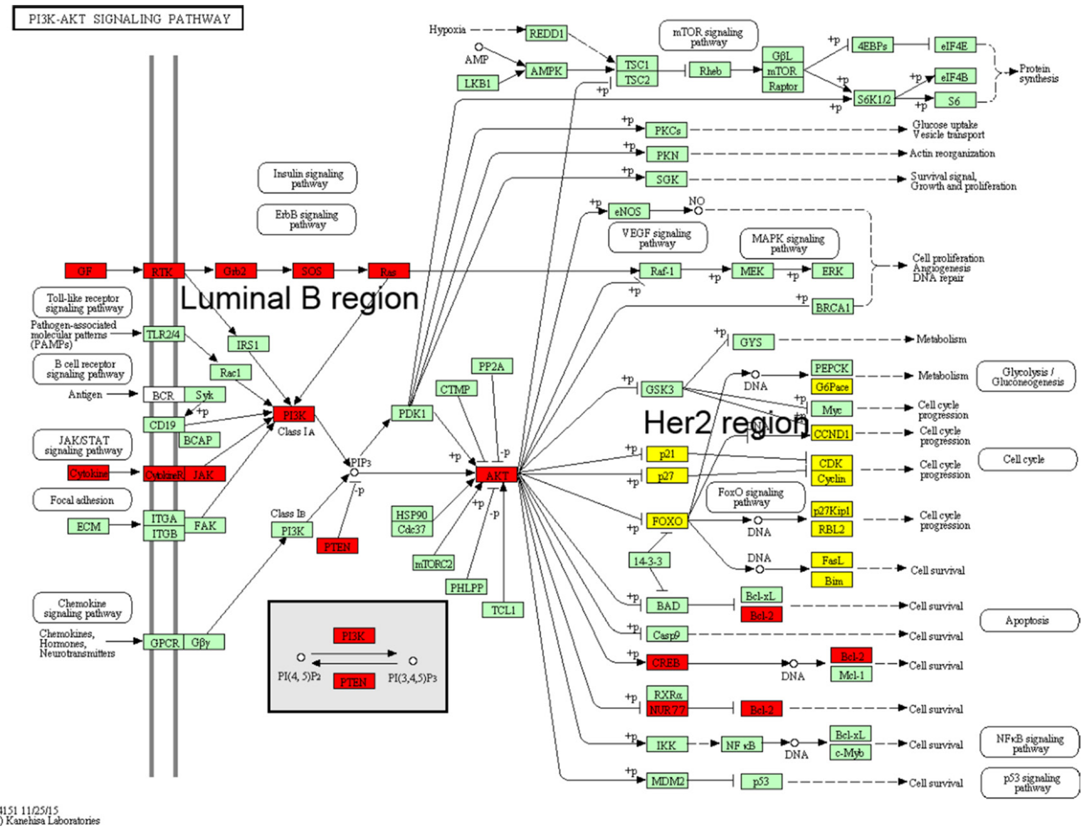

Supplementary Figure 4: Two subpathway regions identified within PI3k-AKT signaling pathway for luminal B and HER2 subtypes. Nodes for PCGs within the luminal B-associated subpathway region are marked in red, and nodes belonging to the HER2-associated subpathway region are marked in yellow.

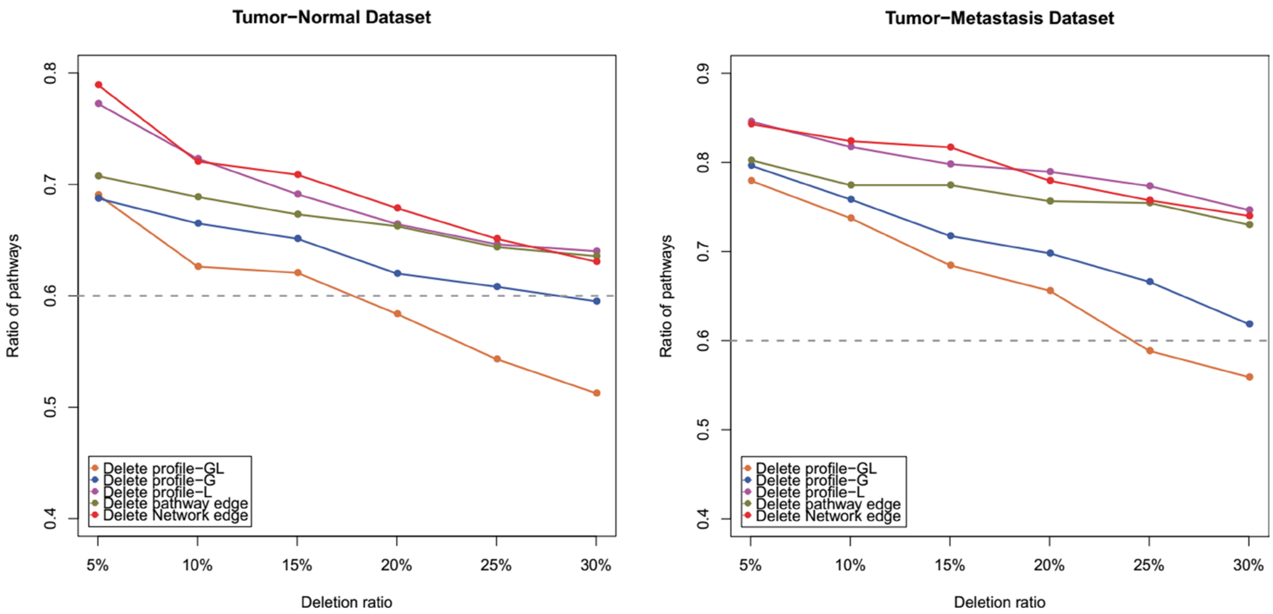

Supplementary Figure 5: Mean ratios for the top 20 subpathways recalled after randomly deleting either *n*% of the lncRNAs (PCGs) from the expression profiles, *n*% of the edges within the pathways, or *n*% of the edges from the lncRNA-PCG association network; *n* varied from 5 to 30 in increments of 5. Left: robustness analysis based on the tumor and normal subset of SRP029880. Right: robustness analysis based on the tumor and metastasis subset of SRP029880. Deletion profile GL: the lncRNA and PCG profiles were simultaneously deleted; Deletion profile G: only the PCG profile was deleted; Deletion profile L: only the lncRNA profile was deleted.

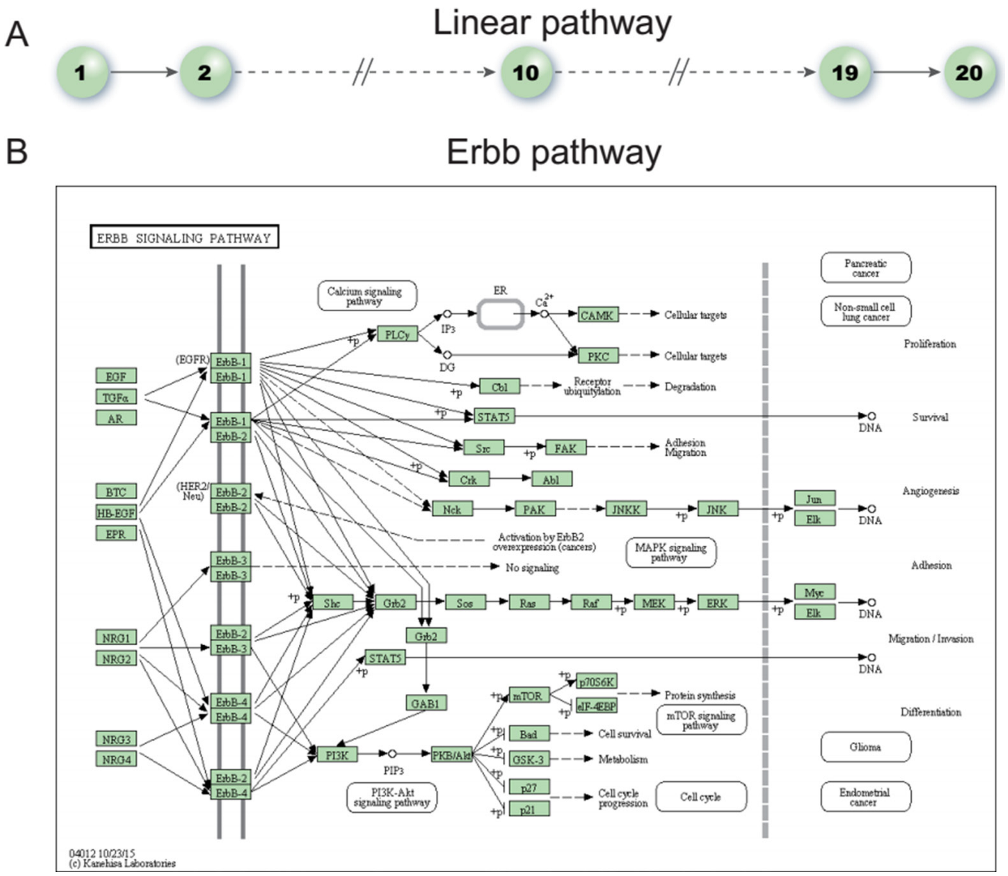

Supplementary Figure 6: Pathway models used in simulation studies. (**A**) Linear pathway structure model with 20 nodes/genes, each of which is associated with one gene. (**B**) ERBB pathway structure model; the interaction of nodes/genes is the same as the ERBB signaling pathway in the KEGG database.

Supplementary Table 1: Dysregulated subpathways that associated with risk lncRNAs in colorectal cancer (FDR < 0.05)

PathwayID	PathwayName	ComponentNum	*P* valueNode	*P* valueEdge	*P* value combined	FDR combined	Reference
path:00230_1	Purine metabolism	23/23/17	0.477	< 0.001	< 0.001	< 0.001	[3, 4]
path:00240_1	Pyrimidine metabolism	19/17/14	0.91	< 0.001	< 0.001	< 0.001	[5]
path:04014_3	Ras signaling pathway	2/6/2	< 0.001	0.8	< 0.001	< 0.001	[6–8]
path:04015_3	Rap1 signaling pathway	12/10/6	0.294	< 0.001	< 0.001	< 0.001	[9, 10]
path:04015_4	Rap1 signaling pathway	3/4/1	< 0.001	0.659	< 0.001	< 0.001	[9, 10]
path:04020_1	Calcium signaling pathway	23/16/11	0.962	< 0.001	< 0.001	< 0.001	[11, 12]
path:04068_1	FoxO signaling pathway	27/19/12	< 0.001	0.344	< 0.001	< 0.001	[13–16]
path:04070_1	Phosphatidylinositol signaling system	11/4/3	0.215	< 0.001	< 0.001	< 0.001	[17, 18]
path:04110_1	Cell cycle	36/34/14	< 0.001	0.064	< 0.001	< 0.001	[19, 20]
path:04115_1	p53 signaling pathway	23/27/13	< 0.001	0.985	< 0.001	< 0.001	[21–23]
path:04141_1	Protein processing in endoplasmic reticulum	11/9/4	< 0.001	1	< 0.001	< 0.001	–
path:04151_1	PI3K-Akt signaling pathway	73/47/25	< 0.001	0.102	< 0.001	< 0.001	[24–26]
path:04151_3	PI3K-Akt signaling pathway	12/9/6	0.959	< 0.001	< 0.001	< 0.001	[24–26]
path:04380_2	Osteoclast differentiation	2/2/1	< 0.001	0.843	< 0.001	< 0.001	–
path:04380_3	Osteoclast differentiation	9/5/4	0.192	< 0.001	< 0.001	< 0.001	–
path:04510_1	Focal adhesion	22/14/9	0.768	< 0.001	< 0.001	< 0.001	[27, 28]
path:04510_2	Focal adhesion	7/9/3	< 0.001	0.379	< 0.001	< 0.001	[27, 28]
path:04620_1	Toll-like receptor signaling pathway	8/9/5	< 0.001	0.88	< 0.001	< 0.001	[29, 30]
path:04630_2	Jak-STAT signaling pathway	16/10/7	0.03	< 0.001	< 0.001	< 0.001	[31–33]
path:04728_2	Dopaminergic synapse	3/3/2	0.002	0.007	1.40E–05	0.00071	–
path:04390_2	Hippo signaling pathway	8/9/6	0.188	0.001	0.000188	0.0093	[34, 35]
path:04012_2	ErbB signaling pathway	2/2/1	0.001	0.451	0.000451	0.0216	[36, 37]
path:04723_1	Retrograde endocannabinoid signaling	14/9/8	0.477	0.001	0.000477	0.0222	–
path:04064_1	NF-kappa B signaling pathway	13/17/9	0.001	0.781	0.000781	0.0354	[38,39]
path:04920_1	Adipocytokine signaling pathway	4/6/3	0.002	0.462	0.000924	0.0407	–
path:04621_1	NOD-like receptor signaling pathway	18/11/7	0.001	0.973	0.000973	0.0418	[40, 41]
path:04917_2	Prolactin signaling pathway	17/15/9	0.004	0.251	0.00100	0.0420	[42]

Supplementary Table 2: Dysregulated subpathways that associated with risk lncRNAs in breast cancer Luminal Asubtype (FDR < 0.05)

PathwayID	Pathway Name	Component Num	*P* value Node	*P* value Edge	*P* value combined	FDR combined
path:00040_1	Pentose and glucuronate interconversions	3/2/1	< 0.001	0.172	< 0.001	< 0.001
path:00053_1	Ascorbate and aldarate metabolism	3/2/1	< 0.001	0.837	< 0.001	< 0.001
path:00230_2	Purine metabolism	8/7/6	0.844	< 0.001	< 0.001	< 0.001
path:00860_1	Porphyrin and chlorophyll metabolism	7/4/2	< 0.001	0.425	< 0.001	< 0.001
path:04014_2	Ras signaling pathway	18/23/10	0.417	< 0.001	< 0.001	< 0.001
path:04015_1	Rap1 signaling pathway	41/37/19	0.244	< 0.001	< 0.001	< 0.001
path:04020_1	Calcium signaling pathway	15/15/12	0.171	< 0.001	< 0.001	< 0.001
path:04110_3	Cell cycle	15/23/6	0.006	< 0.001	< 0.001	< 0.001
path:04151_4	PI3K-Akt signaling pathway	4/16/3	< 0.001	0.731	< 0.001	< 0.001
path:04310_1	Wnt signaling pathway	35/37/21	0.486	< 0.001	< 0.001	< 0.001
path:04380_2	Osteoclast differentiation	14/20/6	< 0.001	0.957	< 0.001	< 0.001
path:04510_1	Focal adhesion	22/25/14	0.74	< 0.001	< 0.001	< 0.001
path:04510_4	Focal adhesion	38/54/22	< 0.001	0.026	< 0.001	< 0.001
path:04630_1	Jak-STAT signaling pathway	33/40/21	0.297	< 0.001	< 0.001	< 0.001
path:04915_1	Estrogen signaling pathway	26/36/17	< 0.001	0.2	< 0.001	< 0.001
path:04144_1	Endocytosis	17/24/9	0.017	0.004	6.80E–05	0.00378
path:04014_3	Ras signaling pathway	4/3/1	0.022	0.015	0.00033	0.0177
path:00983_2	Drug metabolism - other enzymes	3/2/1	0.002	0.173	0.000346	0.0179
path:04151_5	PI3K-Akt signaling pathway	10/14/7	0.359	0.001	0.000359	0.0179
path:00140_1	Steroid hormone biosynthesis	4/3/2	0.009	0.059	0.000531	0.0256
path:04540_2	Gap junction	11/15/6	0.003	0.191	0.000573	0.0267

Supplementary Table 3: Dysregulated subpathways that associated with risk lncRNAs in breast cancer Luminal B subtype (FDR < 0.05)

PathwayID	PathwayName	ComponentNum	*P* valueNode	*P* valueEdge	*P* valuecombined	FDRcombined
path:04015_3	Rap1 signaling pathway	12/13/9	0.38	< 0.001	< 0.001	< 0.001
path:04110_2	Cell cycle	18/28/7	< 0.001	0.674	< 0.001	< 0.001
path:04151_1	PI3K-Akt signaling pathway	30/28/14	< 0.001	0.82	< 0.001	< 0.001
path:04350_1	TGF-beta signaling pathway	21/23/11	< 0.001	0.951	< 0.001	< 0.001
path:04380_2	Osteoclast differentiation	3/13/3	< 0.001	0.977	< 0.001	< 0.001
path:04510_4	Focal adhesion	6/6/1	< 0.001	0.747	< 0.001	< 0.001
path:04664_2	Fc epsilon RI signaling pathway	7/8/1	< 0.001	0.938	< 0.001	< 0.001
path:04919_1	Thyroid hormone signaling pathway	5/8/1	< 0.001	0.498	< 0.001	< 0.001
path:04110_3	Cell cycle	10/19/6	0.002	0.012	2.40E–05	0.00341

Supplementary Table 4: Dysregulated subpathways that associated with risk lncRNAs in breast cancer Her2 subtype (FDR < 0.05)

PathwayID	PathwayName	ComponentNum	*P* valueNode	*P* valueEdge	*P* valuecombined	FDRcombined
path:00140_1	Steroid hormone biosynthesis	11/8/7	0.684	< 0.001	< 0.001	< 0.001
path:00380_1	Tryptophan metabolism	5/3/2	0.524	< 0.001	< 0.001	< 0.001
path:00591_1	Linoleic acid metabolism	3/1/1	0.927	< 0.001	< 0.001	< 0.001
path:04020_1	Calcium signaling pathway	24/24/17	0.738	< 0.001	< 0.001	< 0.001
path:04068_1	FoxO signaling pathway	43/75/18	< 0.001	0.043	< 0.001	< 0.001
path:04110_2	Cell cycle	37/64/14	< 0.001	0.004	< 0.001	< 0.001
path:04115_1	p53 signaling pathway	24/52/11	< 0.001	0.999	< 0.001	< 0.001
path:04150_2	mTOR signaling pathway	2/15/2	< 0.001	0.638	< 0.001	< 0.001
path:04151_6	PI3K-Akt signaling pathway	15/45/7	< 0.001	0.144	< 0.001	< 0.001
path:04151_7	PI3K-Akt signaling pathway	15/21/7	0.201	< 0.001	< 0.001	< 0.001
path:04510_6	Focal adhesion	3/18/1	< 0.001	0.002	< 0.001	< 0.001
path:04530_6	Tight junction	4/7/1	< 0.001	0.935	< 0.001	< 0.001
path:04922_1	Glucagon signaling pathway	19/20/14	0.816	< 0.001	< 0.001	< 0.001
path:04530_5	Tight junction	2/1/1	0.145	0.003	0.000435	0.0286
path:04917_3	Prolactin signaling pathway	6/18/3	0.016	0.027	0.000432	0.0286
path:00230_2	Purine metabolism	2/10/2	0.002	0.284	0.000568	0.0345
path:04015_2	Rap1 signaling pathway	14/18/11	0.564	0.001	0.000564	0.0345
path:04261_1	Adrenergic signaling in cardiomyocytes	27/23/14	0.06	0.01	6.00E–04	0.0351
path:00980_1	Metabolism of xenobiotics by cytochrome P450	8/7/6	0.714	0.001	0.000714	0.0403
path:04713_1	Circadian entrainment	27/28/19	0.163	0.005	0.000815	0.0444
path:04510_4	Focal adhesion	14/34/9	0.148	0.006	0.000888	0.0468

Supplementary Table 5: Dysregulated subpathways that associated with risk lncRNAs in breast cancer basal-like subtype (FDR < 0.05)

PathwayID	PathwayName	ComponentNum	*P* valueNode	*P* valueEdge	*P* valuecombined	FDRcombined
path:00230_1	Purine metabolism	22/32/17	0.703	< 0.001	< 0.001	< 0.001
path:00500_2	Starch and sucrose metabolism	13/7/3	0.748	< 0.001	< 0.001	< 0.001
path:04014_3	Ras signaling pathway	17/36/9	0.137	< 0.001	< 0.001	< 0.001
path:04015_5	Rap1 signaling pathway	5/13/3	0.091	< 0.001	< 0.001	< 0.001
path:04020_2	Calcium signaling pathway	9/11/8	0.672	< 0.001	< 0.001	< 0.001
path:04024_6	cAMP signaling pathway	2/13/1	< 0.001	0.688	< 0.001	< 0.001
path:04062_1	Chemokine signaling pathway	29/35/17	0.526	< 0.001	< 0.001	< 0.001
path:04068_2	FoxO signaling pathway	21/86/9	< 0.001	0.474	< 0.001	< 0.001
path:04068_3	FoxO signaling pathway	5/13/3	< 0.001	0.764	< 0.001	< 0.001
path:04110_1	Cell cycle	34/98/11	< 0.001	0.025	< 0.001	< 0.001
path:04115_1	p53 signaling pathway	35/92/16	< 0.001	1	< 0.001	< 0.001
path:04151_1	PI3K-Akt signaling pathway	46/121/15	< 0.001	0.971	< 0.001	< 0.001
path:04151_3	PI3K-Akt signaling pathway	16/33/8	0.078	< 0.001	< 0.001	< 0.001
path:04151_6	PI3K-Akt signaling pathway	14/23/9	0.568	< 0.001	< 0.001	< 0.001
path:04310_3	Wnt signaling pathway	10/47/7	< 0.001	0.219	< 0.001	< 0.001
path:04510_1	Focal adhesion	77/145/30	< 0.001	< 0.001	< 0.001	< 0.001
path:04512_1	ECM-receptor interaction	39/48/17	0.668	< 0.001	< 0.001	< 0.001
path:04630_1	Jak-STAT signaling pathway	31/67/16	0.258	< 0.001	< 0.001	< 0.001
path:04917_1	Prolactin signaling pathway	13/55/8	< 0.001	0.778	< 0.001	< 0.001
path:04014_4	Ras signaling pathway	2/21/2	0.002	0.146	0.000292	0.0184
path:04919_2	Thyroid hormone signaling pathway	33/93/18	0.002	0.25	5.00E–04	0.0304
path:04144_1	Endocytosis	9/18/5	0.103	0.008	0.000824	0.0484

Supplementary Table 6: The detail information of 28 RNA-Seq datasets used for construction lncRNA-mRNA association network

Index	Sample number	SRA accession ID
1	10	ERP000418
2	31	SRP005408
3	64	ERP000546
4	20	SRP002079
5	11	SRP005411
6	29	ERP000550
7	8	SRP006676
8	10	ERP000573
9	30	SRP002628
10	11	SRP006731
11	12	ERP000710
12	8	SRP003611
13	16	SRP007338
14	18	ERP000992
15	7	SRP003767
16	9	SRP007494
17	6	SRP000302
18	6	SRP004879
19	16	SRP010166
20	6	SRP000626
21	8	SRP004903
22	31	SRP010280
23	16	SRP000727
24	26	SRP005169
25	10	SRP010483
26	6	SRP001119
27	21	SRP005242
28	11	SRP013224
